# Metformin activates KDM2A to reduce rRNA transcription and cell proliferation by dual regulation of AMPK activity and intracellular succinate level

**DOI:** 10.1038/s41598-019-55075-0

**Published:** 2019-12-10

**Authors:** Yuji Tanaka, Akimitsu Konishi, Hideru Obinata, Makoto Tsuneoka

**Affiliations:** 1 Laboratory of Molecular and Cellular Biology, Faculty of Pharmacy, Takasaki University of Health and Welfare, Takasaki, Japan; 2 Department of Biochemistry, Gunma University Graduate School of Medicine, Maebashi, Japan; 3 Education and Research Support Center, Gunma University Graduate School of Medicine, Maebashi, Japan

**Keywords:** Epigenetics, Transcription

## Abstract

Metformin is used to treat type 2 diabetes. Metformin activates AMP-activated kinase (AMPK), which may contribute to the action of metformin. Metformin also shows anti-proliferation activity. However, the mechanism is remained unknown. We found that treatment of MCF-7 cells with metformin induced the demethylase activity of KDM2A in the rDNA promoter, which resulted in reductions of rRNA transcription and cell proliferation. AMPK activity was required for activation of KDM2A by metformin. Because demethylase activities of JmjC-type enzymes require a side reaction converting α-ketoglutarate to succinate, these organic acids may affect their demethylase activities. We found that metformin did not induce KDM2A demethylase activity in conditions of a reduced level of α-ketoglutarate. A four-hour treatment of metformin specifically reduced succinate, and the replenishment of succinate inhibited the activation of KDM2A by metformin, but did not inhibit the activation of AMPK. Metformin reduced succinate even in the conditions suppressing AMPK activity. These results indicate that metformin activates AMPK and reduces the intracellular succinate level, both of which are required for the activation of KDM2A to reduce rRNA transcription. The results presented here uncover a novel factor of metformin actions, reduction of the intracellular succinate, which contributes to the anti-proliferation activity of metformin.

## Introduction

Metformin is used to treat people with type 2 diabetes^1–4^. It has been reported that metformin inhibits complex I activity^5,6^. This inhibition results in changes in some metabolite levels and reduction of ATP production which leads to activation of AMP-activated kinase (AMPK), a key regulator for energy homeostasis. AMPK regulates various metabolisms including gluconeogenesis^2,7–9^, which may contribute to the therapeutic activity of metformin. Metformin was also reported to have anti-proliferation activities in various types of cancers including breast cancers^2,10–12^. However, the mechanism of the anti-proliferation activities of metformin remains unclear.

The ribosome is a unique machine to synthesize proteins in organisms^13,14^, and the control of ribosome biogenesis is a critical factor in the regulation of cellular activities^15–18^. Because the level of rRNA transcription is a major factor determining the production of ribosomes, the regulation of rRNA transcription affects multiple cellular activities, including cell proliferation^13,14,16,18–23^. Recently, increasing numbers of studies have shown that epigenetic regulators control rRNA transcription^13,14,23–27^. We previously showed that a JmjC histone demethylase, lysine-demethylase 2 A (KDM2A), decreases the dimethylated lysine 36 of histone H3 (H3K36me2) in the ribosome RNA gene (rDNA) promoter and represses rRNA transcription under starvation in breast cancer cells^28,29^. We also found that glucose depletion activates AMPK, and then KDM2A to reduce rRNA transcription and cell proliferation of breast cancer cells^28,30^.

The activities of JmjC enzymes including KDM2A require α-ketoglutarate (α-KG), which is converted to succinate during their demethylation processes^31–33^. It was reported that the intracellular levels of α-KG and succinate can affect the activities of JmjC enzymes^28,34–37^. For example, a defective mutation of succinate dehydrogenase, a TCA cycle enzyme, contributes to tumorigenesis in a subset of human cancers. This could be due to changes in the succinate level and subsequent epigenetic reprograming by JmjC enzymes^35,38^. We previously reported that a cell-permeable succinate, dimethyl-succinate (DMS), inhibited the demethylation activity of KDM2A and the reduction of rRNA transcription induced by serum and glucose starvation^28^.

Here, we investigated whether metformin controlled the KDM2A activity to regulate rRNA transcription. The effects of α-KG and succinate on the activation of KDM2A were also examined. We found that metformin activated KDM2A to reduce rRNA transcription and cell proliferation. Interestingly, a four-hour treatment with metformin not only activated AMPK, but also independently decreased the intracellular succinate level; metformin activated AMPK in conditions without reduction of the succinate level and reduced the level of intracellular succinate in conditions inhibiting AMPK activity. The activation of KDM2A required both the AMPK activity and the decrease of the intracellular succinate level. Our results indicate that reduction of the intracellular succinate level is a novel mechanism by which metformin activates KDM2A and exerts its anti-proliferation activity.

## Results

### Metformin induces KDM2A activity to reduce rRNA transcription

**Figure 1:**
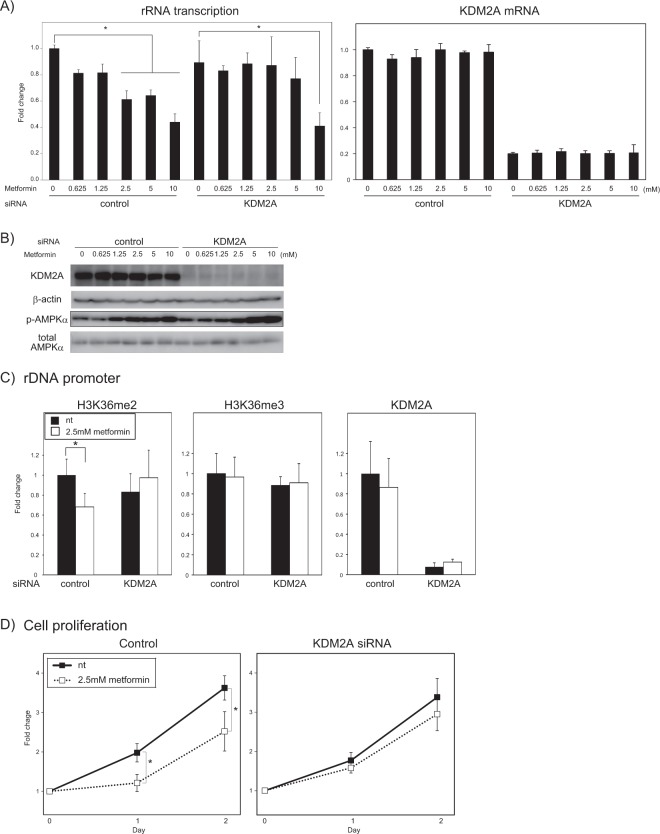
KDM2A-dependent reduction of rRNA transcription by metformin. (**A**) KDM2A-dependent reduction of the rRNA transcription induced by metformin. MCF-7 cells transfected with control siRNA or siRNA for KDM2A were cultured with metformin at indicated concentrations for 4 h. Total RNAs were isolated and analyzed by quantitative real-time PCR (qRT-PCR) to detect rRNA transcription (pre-rRNA) (left panel) and KDM2A mRNA (right panel). The ratios of the values for cells treated with various conditions to those for cells treated with control siRNA without metformin are shown. (**B**) Activation of AMPK by metformin in KDM2A-knockdown cells. Cells transfected with control siRNA or KDM2A siRNA were cultured with metformin for 4 h. The levels of KDM2A, β-actin, phosphorylated-AMPKα (Thr172) (p-AMPKα) and total AMPKα were analyzed by immunoblotting. The uncropped images are shown in Fig. S8. (**C**) KDM2A-dependent reduction of H3K36me2 marks in the rDNA promoter induced by metformin. MCF-7 cells transfected with control siRNA or KDM2A siRNA were cultured with or without 2.5 mM metformin for 4 h. The levels of H3K36me2, H3K36me3, and KDM2A in the rDNA promoter were analyzed by ChIP assay. The results are expressed as fold changes of the values with various conditions to those in cells cultured with control siRNA without metformin treatment. (**D**) KDM2A-dependent reduction of cell proliferation induced by metformin. MCF-7 cells transfected with control siRNA or KDM2A siRNA were cultured with or without 2.5 mM metformin for 2 days. On the indicated days, cells were counted, and the results are shown as the fold change of cell numbers on the indicated day to those at day 0. All experiments were performed three times (n = 3), and the mean values with standard deviations are indicated. *P < 0.05.

Breast cancer cell line MCF-7 cells were treated with metformin to examine whether metformin activated KDM2A to reduce rRNA transcription. Treatment of cells with metformin for four hours reduced rRNA transcription in a dose-dependent manner (Fig. 1A). A KDM2A knockdown impaired the reductions when cells were treated with 2.5 and 5 mM metformin (Fig. 1A). The KDM2A knockdown did not impair the activation of AMPK by metformin (Fig. 1B). We previously reported that KDM2A reduces the levels of H3K36me2 specifically in the rDNA promoter under starvation conditions, but does not reduce them in the transcribed region^28,30^. The levels of H3K36me3, which is not a direct substrate for KDM2A^32^, are not reduced under starvation conditions in both the rDNA promoter and the transcribed regions^28^. The treatment with 2.5 mM metformin reduced the level of H3K36me2 but not H3K36me3 in the rDNA promoter (Fig. 1C). A knockdown of KDM2A impaired the reduction of H3K36me2 by metformin (Fig. 1C). In the transcribed region, reductions of H3K36me2 and H3K36me3 marks by metformin were not observed (Fig. S1). We previously showed that the KDM2A-dependent reduction of rRNA transcription by starvation reduces proliferation of breast cancer cells^30^. It was found that treatment with 2.5 mM metformin reduced cell proliferation, and the KDM2A knockdown suppressed the reduction (Fig. 1D).Together these results suggest that metformin activates KDM2A to reduce H3K36me2 in the rDNA promoter and rRNA transcription, which results in a reduction of cell proliferation.

Previously, it was shown that the AMPK activation is required for the activation of KDM2A to reduce rRNA transcription in response to glucose starvation^30^. The AMPK inhibitor compound C and a new AMPK inhibitor SBI-0206965^39^, which shows lower kinase promiscuity than compound C, inhibited AMPK activity and the reduction of rRNA transcription induced by metformin (Fig. S2A–D). A knockdown of AMPKα also inhibited the reduction of rRNA transcription by metformin (Fig. S2E,F). Compound C and SBI-0206965 also impaired the metformin-induced reduction of H3K36me2 in the rDNA promoter (Fig. S2G,H). These results suggest that AMPK activity is required for the metformin-induced reduction of rRNA transcription and H3K36me2 level in the rDNA promoter through KDM2A.

### Treatments that decrease α-KG level inhibit the activation of KDM2A by metformin

The reaction of the JmjC-type demethylases proceeds with a side reaction that produces succinate from α-KG^32^. Therefore, α-KG and succinate may affect their demethylase activities. It was reported that treatment with a cell-permeable α-KG, dimethyl-alpha ketoglutarate (DMαKG), induces JmjC-enzyme activities^36,40^. We found that DMαKG treatment induced the KDM2A demethylase activity in rDNA promoter and reduced rRNA transcription (Fig. S3).

**Figure 2:**
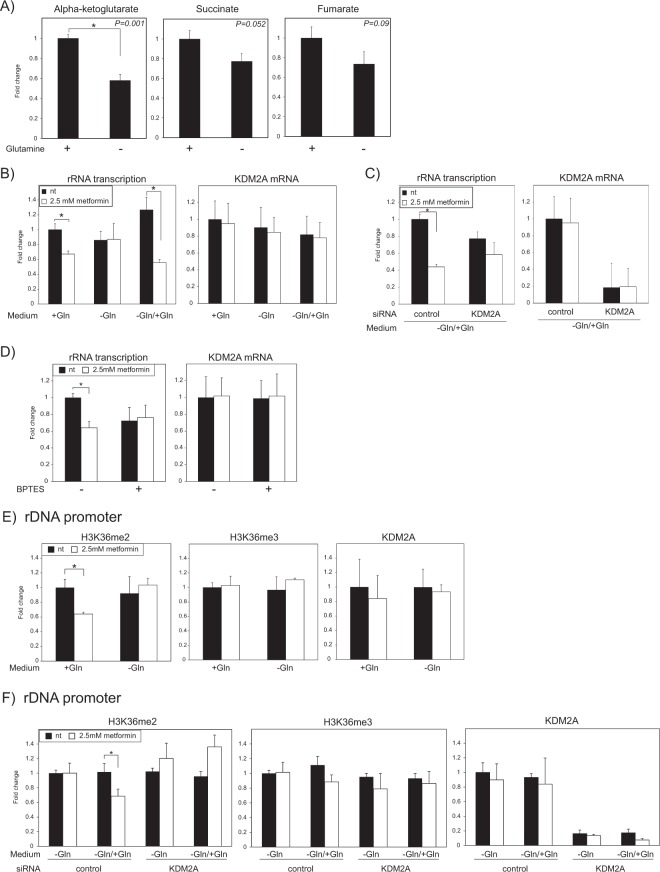
Treatments that decrease α-KG level inhibit the activation of KDM2A by metformin. (**A**) Reduction of α-KG levels in cells cultured in glutamine-free medium. MCF-7 cells were cultured in glutamine-free or -containing medium for 4 h. The metabolites in cells were analyzed by LC-MS/MS. The levels of α-KG, succinate, and fumarate in cells cultured in glutamine-free medium (−Gln) are shown as ratios to those in glutamine-containing medium (+Gln). The *p*-values are shown at the upper right in each panel. (**B**) Glutamine-depletion inhibits the reduction of rRNA transcription by metformin. MCF-7 cells were treated with 2.5 mM metformin for 4 h in glutamine-containing medium (+Gln), -free (−Gln) medium, or -free medium with added glutamine (−Gln/+Gln). The total RNAs were isolated from cells and analyzed by qRT-PCR to detect rRNA transcription (pre-rRNA) and KDM2A mRNA. The ratios of the values in various conditions to those with glutamine (+Gln) without metformin are shown. (**C**) KDM2A-dependent reduction of rRNA transcription induced by metformin in glutamine-free medium with glutamine. MCF-7 cells transfected with control siRNA or siRNA for KDM2A were cultured with or without 2.5 mM metformin for 4 h in glutamine-free medium added glutamine (−Gln/+Gln). The levels of rRNA transcription (left) and KDM2A mRNA (right) were detected by qRT-PCR. The ratios of the values in various condition to those with control siRNA without metformin are shown. (**D**) A glutaminase inhibitor, BPTES, impairs the reduction of rRNA transcription induced by metformin. MCF-7 cells were pre-cultured with or without 2 μM BPTES for 16 h and treated with 2.5 mM metformin for 4 h. The levels of rRNA transcription and KDM2A mRNA were detected by qRT-PCR. The fold changes of the values in various conditions to those without both BPTES and metformin are shown. (**E**) Glutamine-depletion inhibits metformin-induced reduction of H3K36me2 in the rDNA promoter. MCF-7 cells were treated with 2.5 mM metformin for 4 h in glutamine-containing medium (+Gln) or glutamine-free medium (−Gln). The levels of H3K36me2, H3K36me3, and KDM2A in the rDNA promoter were analyzed by ChIP assay. The fold changes of the values in various conditions to glutamine-containing medium without metformin are shown. (**F**) KDM2A- and Gln-dependent reduction of H3K36me2 in the rDNA promoter induced by metformin. MCF-7 cells transfected with control siRNA or KDM2A siRNA were cultured with or without 2.5 mM metformin for 4 h in glutamine-free medium (−Gln) or glutamine-free medium with added glutamine (−Gln/+Gln). The levels of H3K36me2, H3K36me3 and KDM2A in the rDNA promoter were detected by ChIP assay. The fold changes of the values in various conditions to those in cells treated with control siRNA, without glutamine (–Gln) and without metformin are shown. All experiments were performed three times (n = 3), and the mean values with standard deviations are indicated. *P < 0.05.

Next, we tried to decrease the levels of intracellular α-KG and evaluated the contribution of α-KG to the activation of KDM2A by metformin. α-KG is generated through two production systems, glycolysis followed by the TCA cycle and glutaminolysis from glutamine. Generally, in cancer cells, the production of α-KG by glutaminolysis is elevated, because cancer cells favor aerobic glycolysis and glucose is often diverted to lactate production^41–43^. When MCF-7 cells were cultured in glutamine-free medium for four hours, the α-KG level was decreased to half (Fig. 2A), and the succinate and fumarate levels tended to be decreased (Fig. 2A). The four-hour metformin treatment did not reduce rRNA transcription in the glutamine-free medium (Fig. 2B, −Gln), but it reduced the rRNA transcription culturing in glutamine-containing medium (Fig. 2B +Gln) and the glutamine-free medium that had been added glutamine (Fig. 2B, −Gln/+Gln). The reduction of rRNA transcription during culturing in the glutamine-free medium that had been added glutamine was inhibited by KDM2A knockdown (Fig. 2C). Pretreatment with an inhibitor of the glutaminase^44^, which inhibits the production of α-KG from glutamine, impaired the reduction of rRNA transcription by metformin (Fig. 2D). The effect of this inhibitor on the metformin-mediated regulation of rRNA transcription was not observed in the glutamine-free medium (Fig. S4). Therefore, two treatments that reduced intracellular α-KG levels inhibited KDM2A activity and the results are continuity of the story that the α-KG level in cells affects the KDM2A activity to reduce rRNA transcription induced by metformin.

Metformin reduced the H3K36me2 levels in the rDNA promoter in glutamine-containing medium but not in glutamine-free medium (Fig. 2E, −Gln). The addition of glutamine to the glutamine-free medium restored the reduction of the H3K36me2 levels induced by metformin (Fig. 2F, −Gln/+Gln). A KDM2A knockdown impaired the reduction of H3K36me2 levels (Fig. 2F). These results suggest that glutamine, which is converted to α-KG, is a critical factor for KDM2A activated by metformin to demethylate H3K36me2 marks in the rDNA promoter.

The rRNA transcription tended to be reduced by culture in glutamine-free medium for four hours without metformin (Fig. 2B). The glutamine-depletion activated AMPK (Fig. S5A) but did not reduce the level of H3K36me2 in the promoter (Fig. 2E) and in the transcribed region (Fig. S5B) of rDNA. These results suggest that the tendency of glutamine-free medium to reduce rRNA transcription occurred independently of the demethylase activity of KDM2A. These results suggest that AMPK activation under glutamine-free medium is not enough to induce the demethylase activity of KDM2A in the rDNA promoter and that the intracellular level of α-KG may be a factor to activate KDM2A.

### Metformin decreases cellular succinate

**Figure 3:**
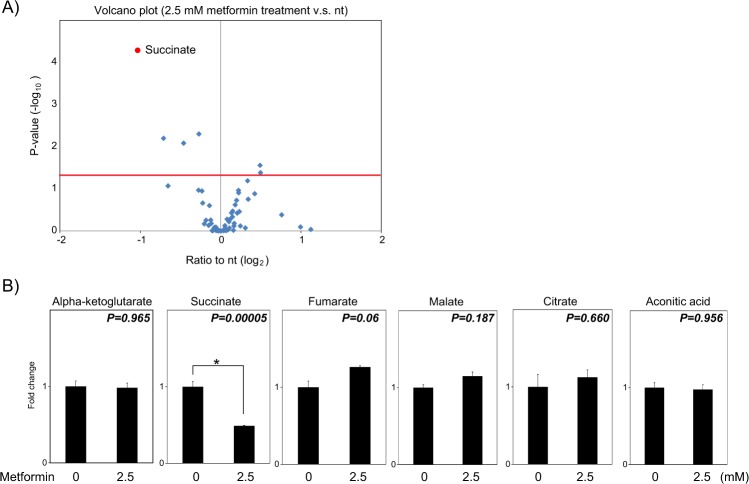
Metformin treatment for 4 hours decreases the succinate level in cells. (**A**) Volcano plot of metabolites in cells treated with 2.5 mM metformin. MCF-7 cells were treated with or without 2.5 mM metformin for 4 h. The metabolites in cells were analyzed by LC-MS/MS. The results are shown as a volcano plot of the ratios and *p*-values calculated from results of 2.5 mM metformin treatment and non-treatment. The value for succinate is drawn as a red circle. The red line indicates where *p* = 0.05 with points above the line having *p* < 0.05. The details of these data were shown in Supplementary Table 1. (**B**) The levels of TCA cycle intermediates in metformin-treated cells. The fold changes of the levels of α-KG, succinate, fumarate, malate, citrate, and aconitic acid in cells treated with 2.5 mM metformin compared to those in untreated cells are shown. The *p*-values are shown at the upper-right in each panel. All experiments were performed three times (n = 3). *P < 0.05.

Recently, it was reported that metformin treatment for more than 24 h decreased the intracellular levels of several metabolites in the TCA cycle^45–47^, including α-KG and succinate. However, the changes of metabolites soon after the metformin treatment have not been reported yet. Thus, we detected the amounts of intracellular metabolites after the metformin treatment in our conditions. After cells were treated with 0, 2.5, or 10 mM metformin for four hours, the levels of 65 metabolites were quantitated by LC-MS/MS (Supplementary Table 1, Figs. 3 and S6). The metabolites changed by metformin with statistical significance are extracted in Supplementary Table 2. Four metabolites were decreased, and two metabolites were increased in cells treated with 2.5 mM metformin, while the seven metabolites were decreased and 22 metabolites were increased in cells treated with 10 mM metformin (Supplementary Table 2).

Interestingly, the level of succinate was apparently most affected by metformin among the metabolites (Figs. 3A and S6A). The TCA cycle intermediates measured in this study are shown in Figs. 3B and S6B. Succinate was decreased to half by 2.5 mM metformin compared to that in non-treated cells (Fig. 3B). The levels of fumarate and malate tended to be slightly increased, while the levels of α-KG, citrate, and aconitic acid were not affected (Fig. 3B). These changes were clearer in cells treated with 10 mM metformin (Fig. S6B). The decrease of succinate as well as the increases of fumarate and malate by metformin were confirmed by another LC-MS/MS measurement with a different LC method (Fig. S6C).

It should be noted that α-KG, which was suggested to activate the KDM2A activity (Figs. 2 and S3), did not increase, and fumarate which was reported to inhibit JmjC enzymes^48^ did not decrease with the metformin treatment (Figs. 3 and S6). Together, these results suggest that succinate, which can work as an inhibitor of JmjC enzymes, was specifically reduced by the four-hour treatment of metformin among TCA cycle intermediates.

Because KDM2A converts α-KG to succinate, the activity of KDM2A would be inhibited by succinate produced by KDM2A. Therefore, the negative feedback loop may exit. On the metformin treatment, KDM2A was activated and succinate was reduced. Therefore, metformin would suppress the effect of the negative feedback loop. One possible way to suppress this loop is that the reduction of succinate by metformin is too quick and large compared to the effect of the negative feedback loop at this time point. The fact that metformin reduced succinate level without change in α-KG level (Fig. 3B) suggest that KDM2A activity on the metformin treatment did not have much effect on the overall levels of the intracellular organic acids, and supported the above possibility.

### Reduction of intracellular succinate is required for KDM2A activation by metformin

**Figure 4:**
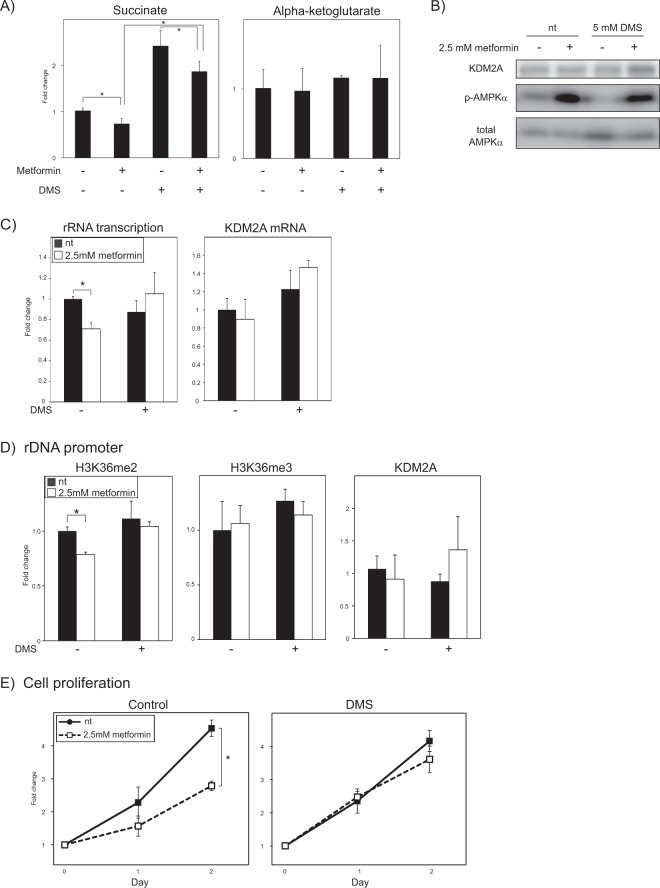
Replenishment of succinate inhibits metformin-induced reductions of rRNA transcription. (**A**) DMS treatment increases the level of succinate in cells treated with metformin. MCF-7 cells were treated with or without 2.5 mM metformin in the presence or absence of 5 mM dimethyl succinate (DMS) for 4 h. Metabolites in cells were analyzed by LC-MS/MS. The fold changes of the levels of succinate and α-KG to those without metformin and DMS are shown. (**B**) DMS does not abolish the AMPK-activation by metformin. MCF-7 cells were cultured with or without 2.5 mM metformin in the presence or absence of 5 mM DMS for 4 h. The levels of KDM2A, phosphorylated-AMPKα (Thr172) (p-AMPKα), and total AMPKα in cells were analyzed by immunoblotting. The uncropped images are shown in Fig. S9. (**C**) DMS inhibits the reduction of rRNA transcription by metformin. MCF-7 cells were cultured for 4 h with or without 2.5 mM metformin in the presence or absence of 5 mM DMS. Total RNAs were isolated, and the levels of rRNA transcription (pre-rRNA) and KDM2A mRNA were measured by qRT-PCR. The fold changes of the values in various conditions compared to those without DMS and metformin are shown. (**D**) DMS inhibits reduction of H3K36me2 marks in the rDNA promoter induced by metformin. MCF-7 cells were cultured for 4 h with or without 2.5 mM metformin in the presence or absence of 5 mM DMS. The levels of H3K36me2, H3K36me3, and KDM2A in the rDNA promoter were analyzed by ChIP assay. The results are expressed as fold changes to the values in various conditions to those without DMS and metformin. (**E**) DMS inhibits the reduction of cell proliferation induced by metformin. MCF-7 cells were cultured with or without 2.5 mM metformin in the presence or absence of 5 mM DMS for 2 days. The fold changes of cell numbers counted at the indicated days compared to those at day 0 are shown. All experiments were performed three times (n = 3), and the mean values with standard deviations are indicated. *P < 0.05.

Our results raised the possibility that the decrease of the succinate level by metformin could contribute to the activation of KDM2A to reduce rRNA transcription. To test this possibility, cells were treated with a cell-permeable succinate, dimethyl succinate (DMS), to increase the intracellular succinate level. The increase of the intracellular succinate was confirmed at four hours in metformin-treated cells with the treatment of 5 mM DMS (Fig. 4A), but it did not abolish the activation of AMPK by metformin (Fig. 4B). The DMS treatment inhibited the reduction of rRNA transcription induced by 2.5 mM metformin for four hours (Fig. 4C). The reduction of the H3K36me2 level in the rDNA promoter induced by metformin was also inhibited by the DMS treatment (Fig. 4D). The levels of H3K36me2 in the rDNA transcribed region (Fig. S7) and H3K36me3 in both the rDNA promoter (Fig. 4D) and the rDNA transcribed regions (Fig. S7) were not changed by metformin or DMS, suggesting the specific effect of DMS on the KDM2A activation in these conditions. The reduction of cell proliferation by metformin was canceled by addition of DMS (Fig. 4E). Together, these results suggest that the reduction of succinate is required for the activation of KDM2A by metformin.

### AMPK activity is dispensable for reduction of succinate induced by metformin

**Figure 5:**
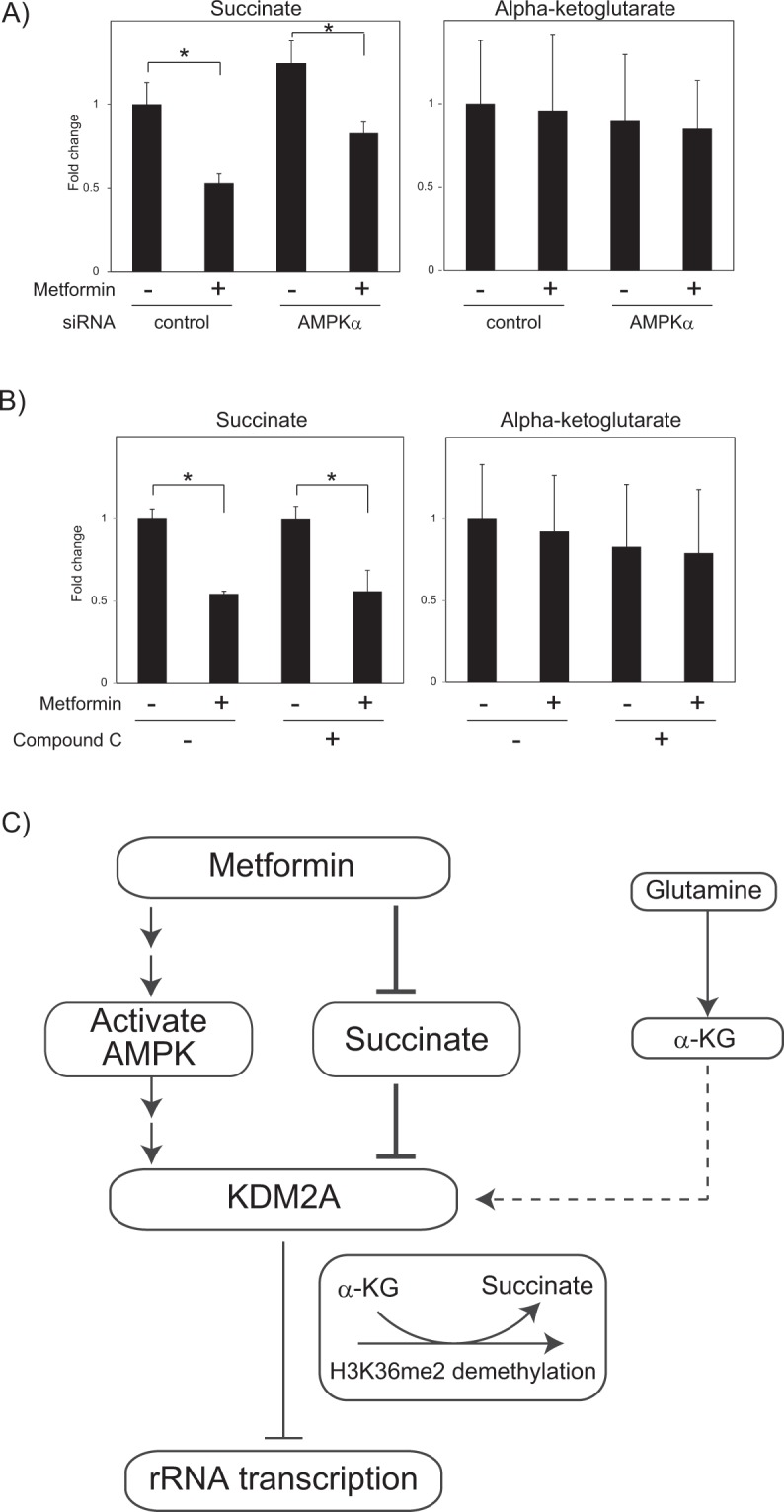
Metformin decreases intracellular succinate level even in conditions suppressing AMPK activity. (**A**) AMPK knockdown does not impair the reduction of succinate level by metformin. MCF-7 cells introduced with control siRNA or siRNA for AMPKα were treated with 2.5 mM metformin for 4 h. Metabolites in these cells were extracted, and the levels of succinate and α-KG were measured by LC-MS/MS. The fold changes of the levels of succinate or α-KG in cells in various conditions compared to those in cells with control siRNA and without metformin are shown. (**B**) Compound C treatment does not impair the reduction of succinate by metformin. MCF-7 cells were pre-cultured in the presence or absence of compound C for 1 h, and then treated with 2.5 mM metformin for 4 h. The levels of succinate and α-KG in these cells were analyzed by LC-MS/MS. The fold changes of the levels of succinate or α-KG in cells in various conditions compared to those in cells in the absence of compound C without metformin are shown. All experiments were performed three times (n = 3), and the mean values with standard deviations are indicated. *P < 0.05. (**C**) A model of KDM2A activation by metformin to regulate rRNA transcription. The metformin treatment induces activation of AMPK and reduction of succinate, both of which mediate to activate KDM2A to repress rRNA transcription. Treatment to reduce the intracellular α-KG level inhibits the KDM2A activation. The levels of both α-KG and succinate influences to the demethylation activity of KDM2A in the rDNA promoter.

Our results suggest that the activation of KDM2A by metformin requires AMPK activation (Figs. 1 and S2) and the reduction of succinate (Fig. 4). Finally, we investigated whether the AMPK activation induced by metformin was required for the decrease of succinate. A knockdown of AMPKα or the AMPK inhibitor compound C (Fig. S2) hardly affected the decrease of succinate by metformin (Fig. 5A,B). These results suggest that the AMPK activity is dispensable for the reduction of the succinate level.

## Discussion

### Metformin induces AMPK activation and reduction of succinate, both of which contribute to activation of KDM2A to reduce rRNA transcription

Metformin induced the H3K36me2-demethylase activity of KDM2A in the rDNA promoter and the reductions of rRNA transcription and cell proliferation. Metformin activated AMPK, and AMPK activity was required for the KDM2A functions to reduce rRNA transcription (Figs. 1 and S2)^30^. Metformin did not induce the KDM2A activity in glutamine-free medium where the intracellular α-KG level was reduced. Metformin treatment for four hours reduced succinate, and the replenishment of succinate with DMS blocked the metformin-mediated KDM2A activation despite AMPK activation (Figs. 2–4). From these results, we propose a mechanism in which the relative amount of α-KG against succinate constitutes a switch inducing the KDM2A activity in the rDNA promoter. Together, our results suggest that metformin induces the activation of AMPK and the reduction of succinate, both of which are required for activation of KDM2A to reduce rRNA transcription (Fig. 5C).

Recently, it was reported that metformin exerted tumor suppressive effects through up-regulation of the ten-eleven translocation 2 (TET2) protein^49^. Wu *et al*. reported that AMPK phosphorylated and stabilized TET2. Because TET2 is a JmjC-type DNA-hydroxymethylase, it is possible that the reduction of succinate by metformin contributes to the activation of TET2. The reduction of succinate by metformin may be widely involved in the anti-proliferation effects of metformin. The metformin dose used in the above tumor model and our study here was higher than that in blood concentration in metformin-treated diabetes patients. Therefore, it is not clear whether the rationale found in the *in vitro* experiments can be applied to the anti-cancer activity of metformin in diabetes patients treated with metformin. Further studies are required to clarify this point.

### Possible mechanisms by which metformin reduces intracellular succinate

Our study is the first report showing the specific reduction of the intracellular succinate level without concomitant reductions of other TCA cycle intermediates including α-KG, fumarate, and malate (Figs. 3 and S6). The reduction of the succinate level occurred even under conditions that suppress AMPK activity (Fig. 5). It had been reported that metformin inhibits complex I activity^5,6^. In addition, recently it was reported that metformin inhibits the redox shuttle enzyme mitochondrial glycerophosphate dehydrogenase (mGPD)^50^. Both complex I and mGPD supply electrons to coenzyme Q (CoQ) through oxidation of NADH or FADH_2_^51^. Therefore, metformin reduces the number of electrons in the electron transfer system. Meanwhile, complex II produces electrons using its succinate dehydrogenase (SDH) activity, which catalyzes the conversion of succinate to fumarate. These electrons are transferred complex III and IV in the electron transport chain to generate ATP. The reduction of electrons by metformin may enforce SDH activity to produce electrons and reduce succinate. According to this hypothesis, an increase of the fumarate level accompanying a decrease of the succinate level would occur, which is consistent to our observation in Figs. 3 and S6.

Our results suggest that the changes in the succinate level in mitochondria control the enzyme activities in the nucleus. There are precedents in which the amount of mitochondrial succinate affects the activities of nuclear factors. A defective mutation of SDH, which increased the succinate level, stabilized hypoxia inducible factor 1 (HIF-1) through inhibition of a JmjC type enzyme HIFα prolyl hydroxylases (PHDs)^34,52^. The mutations in the catalytic sites of SDH also influenced the oxidation of 5-methylcytosine by TET^48^. Recently a mitochondrial dicarboxylate carrier (DIC) SLC25A10 in the inner membrane was suggested to mediate the equilibration of mitochondrial and cytosolic succinate pools in brown adipocytes and macrophage cells^53–56^. These observations suggest the presence of inter-organelle communication between mitochondria and the nucleus, using succinate as a messenger molecule to modulate JmjC enzyme-activities in the nucleus.

Alternatively, it is also possible that the level of succinate is initially decreased in the cytoplasm and/or nuclei by metformin. Recently, numerous metformin-binding proteins were predicted^57^. A JmjC protein KDM6A/UTX was predicted to be a metformin-binding protein, and it was suggested that metformin inhibited its demethylase activity^57^. Because the JmjC enzymes produce succinate in the demethylation process^32^, the inhibition of KDM6A/UTX demethylase activity may reduce the succinate level in the nucleus.

We demonstrated here that the levels of α-KG and succinate are pivotal factors in the regulation of the KDM2A demethylase activity by metformin. Observations of succinate levels inside cells in each organelle would further clarify the regulation mechanism of nuclear enzymes.

## Materials and Methods

### Antibodies

Anti-dimethylated histone H3 lys36 antibody (MAB Institute, Inc.; #MABI0332-100), anti-trimethylated histone H3 lys36 antibody (MAB Institute, Inc.; #MABI0333-100), and anti-histone H3 antibody (Abcam; # ab1791) were purchased. The control antibody (Cell Signaling, normal rabbit IgG; #2729S) for ChIP assays was also purchased. The anti-KDM2A antibody produced in previous study was used^28^. Anti-phosphorylated AMPKα antibody (Thr-172), anti-AMPKα antibody and β-actin antibody for immunoblotting were purchased (AMPK and ACC Antibody Sampler Kit, Cell Signaling; #9957 and Sigma, AC-15; #A5441).

### Cell culture and culture medium

The human breast adenocarcinoma cell line MCF-7 was cultured in RPMI-1640 medium (RPMI, Nakalai Tesque; #30264) supplemented with 10% fetal calf serum (FCS), 100 units/ ml penicillin G (Nakalai Tesque; #26239-42), and 100 µg/ml streptomycin sulfate (Nakalai Tesque; #33204-92). Cells were maintained at 37 °C in humidified atmosphere containing 5% CO_2_.

In the experiments for culturing in glutamine-free medium (−Gln), MCF-7 cells were cultured in RPMI-1640 medium without L-glutamine (RPMI 1640 without L−Gln, Nakalai Tesque; # 05176-25) supplemented with 10% fetal calf serum (FCS), 100 units/ ml penicillin G, and 100 µg/ml streptomycin sulfate. Glutamine (Nakalai Tesque; #16948-04; 200mM-L-Glutamine Stock Solution) was added to glutamine-free medium with the same concentration to that in the standard RPMI-1640 medium (RPMI, Nakalai Tesque; # 30264) to produce glutamine-free medium with added glutamine (−Gln/+Gln).

### Agents

Metformin (TCI; #M2009; metformin hydrochloride), and dimethyl-succinate (DMS) (TCI; #S0104; succinic acid dimethyl ester) were purchased. Metformin was dissolved in distilled water. Compound C (IN Solution™ AMPK Inhibitor, compound C, Calbiochem; #171261), SBI-0206965 (Sigma; #SML1540) and bis-2-(5-phenylacetamido-1,3,4-thiadiazol-2-yl) ethyl sulfide (BPTES) (Merck Millipore; # 530030) were purchased.

### siRNAs

Cells were transfected with siRNAs using Lipofectamine RNAiMAX (Life Technologies; # 13778-150) according to the manufacturer’s instructions with suitable amounts of siRNAs for this work (150 pmol per 10 cm dis’). The siRNA specific for KDM2A was 5′-GAACCCGAAGAAGAAAGGAUUCGUU-3′, which was previously described^28^. The control siRNA, Stealth™ RNAi Negative Control Medium GC Duplex (Life Technologies), was purchased. Three days after transfection, these cells were used for each experiment. For knockdown of AMPKα1/2, the siRNA for AMPKα1/2 (Santa Cruz Biotechnology; #sc-45312) and control siRNA-A (Santa Cruz Biotechnology; #sc-37007) were used as previously described^30^.

### Total RNA extraction and quantitative reverse transcription-polymerase chain reaction (qRT-PCR)

Total RNA was isolated from cells using NucleoSpin RNA II kit (Takara Bio; #U0955C) according to the manufacturer’s instructions. Single-strand cDNA was synthesized from total RNA (0.4 µg) by a Superscript III First-strand Synthesis system (Life Technology; # 18080-051) using random hexamers according to the manufacturer’s instructions. The products were diluted up to 100 µl with distilled water, and 2.5 µl of the resultant single-strand cDNA was used as the template for qRT-PCR using a KAPA SYBR FAST qPCR Master Mix Kit (KAPA Biosystems; # KR0389) with an Mx3000P QPCR system (Agilent Technologies) or CFX Connect Real-Time PCR Detection System (Bio-Rad Laboratories, Inc.). The values measured by qRT-PCR were normalized by the amounts of β-actin mRNA.

To evaluate levels of rRNA transcription, the amounts of pre-rRNA were measured. The sets of PCR primers for amplification of the pre-rRNA were 5′-GCTGACACGCTGTCCTCTG-3′ and 5′-TCGGACGCGCGAGAGAAC-3′ (a sequence in the 5′ untranslated region 1–155 of pre-rRNA); for KDM2A, the primers were 5′-TCCCCACACACATTTTGACATC-3′ and 5′-GGGGTGGCTTGAGAGATCCT-3′; for β-actin, the primers were 5′-CGTCTTCCCCTCCATCGT-3′ and 5′-GAAGGTGTGGTGCCAGATTT-3′ as previously described^30^.

### ChIP assay

The chromatin immunoprecipitation (ChIP) assay was performed as described previously^30^, using Dynabeads protein G (ThermoFisher; #10003D) with buffer components from a ChIP assay kit (Merck Millipore). Cells were fixed with 1% formaldehyde for 10 min at 37 ^o^C. After addition of 0.125 M glycine and incubation for 5 min, cells were washed with PBS three times. Then cells were lysed with ChIP lysis buffer, sonicated, and used for a ChIP assay. The immunoprecipitated DNA was purified by a Chelex-100-based DNA isolation procedure^58^, and the amounts of the DNA fragment were detected by qRT-PCR as described above. The primers used for the detection of rDNA promoter (rDNA from +1 to +155 from the transcriptional start site; the same primers as used for pre-rRNA detection) were described previously^28^. To detect specific binding, the values obtained by specific antibodies were divided by input (% of input) and normalized by the values of the control antibody (normal rabbit IgG). When detecting histone modifications, the values for the specific binding were normalized by the values for H3 (% of specific bound/input normalized by H3). The experiments were repeated at least three times. The averages and standard deviations of the results were shown.

### Cell counting

To detect cell proliferation, cells were counted by using a Burker-Turk type hemocytometer. These cell numbers were averaged from at least three experiments.

### Cell collection for LS-MS/MS analysis

Cells were cultured to semi-confluent in 6 cm dishes. After treatments, cells were washed by PBS three times. Methanol containing 2 μM 2-(N-morpholino) ethanesulfonic acid (MES) as an internal standard was added to dishes, and the dishes were rocked for 15 min at room temperature (RT). The extracts were collected and centrifuged with 15,000 rpm for 15 min at RT. The supernatants were dried by a vacuum evaporator, re-suspended with distilled water, and used for the detection of metabolites by LC-MS/MS.

### Measurement of metabolites by LC-MS/MS

Liquid chromatography-tandem mass spectrometry (LC-MS/MS) was performed by using a LCMS-8050 triple quadrupole mass spectrometer system (Shimadzu, Kyoto, Japan). The relative levels of metabolites in the central metabolic pathways were determined by using the Method Package for Primary Metabolites (Shimadzu) with a Discovery HS F5-3 column (Sigma-Aldrich, St. Louis, MO) according to the manufacturer’s instructions. In some cases, metabolites in TCA cycles were analyzed by a different LC method using a Mastro^TM^ SP column (Shimadzu) for better peak detection. The experiments were performed three times (n = 3). The results were expressed as the ratios with *p*-values of each metabolite of the values from cells under various conditions to those from control cells were calculated.

### Immunoblotting

Cells were harvested after treatment in each experiment and extracted using an SDS-PAGE sampling buffer (4% SDS solution containing 100 mM Tris, pH 6.8, 50 mM DTT, and 20% glycerol) of suitable volume to adjust extracts to the same concentration. Cell extracts were separated by SDS-PAGE and transferred to a PVDF membrane (Millipore, Burlington, MA; #IPVH00010). After treatment with antibodies, bands were detected using an Immobilon Western system (Millipore; #WBKLS0100) as described previously^30^.

### Statistics and reproducibility

Sample sizes and error bars are indicated in each figure legend. The *p*-values from an ANOVA (Tukey test) calculated with EZR on R commander^59^ are shown as an asterisk (*) in the figure.

## Supplementary information

Supplementary information

## Data Availability

All data generated or analysed during this study are included in this published article (and its Supplementary Information files).

## References

[1] Rena G, Pearson ER, Sakamoto K (2013). Molecular mechanism of action of metformin: old or new insights?. Diabetologia.

[2] Pernicova I, Korbonits M (2014). Metformin-mode of action and clinical implications for diabetes and cancer. Nat Rev Endocrinol.

[3] Pryor R, Cabreiro F (2015). Repurposing metformin: an old drug with new tricks in its binding pockets. Biochem J.

[4] Yu X (2017). Anti-tumor activity of metformin: from metabolic and epigenetic perspectives. Oncotarget.

[5] Owen MR, Doran E, Halestrap AP (2000). Evidence that metformin exerts its anti-diabetic effects through inhibition of complex 1 of the mitochondrial respiratory chain. Biochem J.

[6] Andrzejewski S, Gravel SP, Pollak M, St-Pierre J (2014). Metformin directly acts on mitochondria to alter cellular bioenergetics. Cancer Metab.

[7] Foretz M (2010). Metformin inhibits hepatic gluconeogenesis in mice independently of the LKB1/AMPK pathway via a decrease in hepatic energy state. J Clin Invest.

[8] Hardie DG, Ross FA, Hawley SA (2012). AMPK: a nutrient and energy sensor that maintains energy homeostasis. Nat Rev Mol Cell Biol.

[9] Hur KY, Lee MS (2015). New mechanisms of metformin action: Focusing on mitochondria and the gut. J Diabetes Investig.

[10] Zakikhani M, Dowling R, Fantus IG, Sonenberg N, Pollak M (2006). Metformin is an AMP kinase-dependent growth inhibitor for breast cancer cells. Cancer Res.

[11] Barzilai N, Crandall JP, Kritchevsky SB, Espeland MA (2016). Metformin as a Tool to Target Aging. Cell Metab.

[12] Zhang HH, Guo XL (2016). Combinational strategies of metformin and chemotherapy in cancers. Cancer Chemother Pharmacol.

[13] Olson, M. O. J. *The nucleolus*. (Springer), doi:10.1007/978-1-4614-0514-6 (2011).

[14] Tanaka Yuji, Tsuneoka Makoto (2018). Control of Ribosomal RNA Transcription by Nutrients. Gene Expression and Regulation in Mammalian Cells - Transcription Toward the Establishment of Novel Therapeutics.

[15] Thomas G (2000). An encore for ribosome biogenesis in the control of cell proliferation. Nat Cell Biol.

[16] Boisvert FM, van Koningsbruggen S, Navascues J, Lamond AI (2007). The multifunctional nucleolus. Nat Rev Mol Cell Biol.

[17] Boulon S, Westman BJ, Hutten S, Boisvert FM, Lamond AI (2010). The nucleolus under stress. Mol Cell.

[18] Kusnadi EP (2015). Regulation of rDNA transcription in response to growth factors, nutrients and energy. Gene.

[19] Grummt I (2003). Life on a planet of its own: regulation of RNA polymerase I transcription in the nucleolus. Genes Dev.

[20] Russell, J. & Zomerdijk, J. C. The RNA polymerase I transcription machinery. *Biochem Soc Symp*, 203–216 (2006). 10.1042/bss0730203 PMC3858827 16626300

[21] Birch JL, Zomerdijk JC (2008). Structure and function of ribosomal RNA gene chromatin. Biochem Soc Trans.

[22] Drygin D, Rice WG, Grummt I (2010). The RNA polymerase I transcription machinery: an emerging target for the treatment of cancer. Annu Rev Pharmacol Toxicol.

[23] Goodfellow SJ, Zomerdijk JC (2013). Basic mechanisms in RNA polymerase I transcription of the ribosomal RNA genes. Subcell Biochem.

[24] Preuss S, Pikaard CS (2007). rRNA gene silencing and nucleolar dominance: insights into a chromosome-scale epigenetic on/off switch. Biochim Biophys Acta.

[25] McStay Brian, Grummt Ingrid (2008). The Epigenetics of rRNA Genes: From Molecular to Chromosome Biology. Annual Review of Cell and Developmental Biology.

[26] Grummt I, Langst G (2013). Epigenetic control of RNA polymerase I transcription in mammalian cells. Biochim Biophys Acta.

[27] Tanaka Y, Tsuneoka M (2013). Control mechanisms of ribosomal RNA transcription. Seikagaku.

[28] Tanaka Y (2010). JmjC enzyme KDM2A is a regulator of rRNA transcription in response to starvation. EMBO J.

[29] Tanaka Y, Umata T, Okamoto K, Obuse C, Tsuneoka M (2014). CxxC-ZF domain is needed for KDM2A to demethylate histone in rDNA promoter in response to starvation. Cell Struct Funct.

[30] Tanaka Y (2015). Mild Glucose Starvation Induces KDM2A-Mediated H3K36me2 Demethylation through AMPK To Reduce rRNA Transcription and Cell Proliferation. Mol Cell Biol.

[31] Klose RJ, Kallin EM, Zhang Y (2006). JmjC-domain-containing proteins and histone demethylation. Nat Rev Genet.

[32] Tsukada Y (2006). Histone demethylation by a family of JmjC domain-containing proteins. Nature.

[33] Shi Y, Whetstine JR (2007). Dynamic regulation of histone lysine methylation by demethylases. Mol Cell.

[34] Selak MA (2005). Succinate links TCA cycle dysfunction to oncogenesis by inhibiting HIF-alpha prolyl hydroxylase. Cancer Cell.

[35] Kaelin WG, McKnight SL (2013). Influence of metabolism on epigenetics and disease. Cell.

[36] Carey BW, Finley LW, Cross JR, Allis CD, Thompson CB (2015). Intracellular alpha-ketoglutarate maintains the pluripotency of embryonic stem cells. Nature.

[37] Wong CC, Qian Y, Yu J (2017). Interplay between epigenetics and metabolism in oncogenesis: mechanisms and therapeutic approaches. Oncogene.

[38] Smith EH, Janknecht R, Maher LJ (2007). 3rd. Succinate inhibition of alpha-ketoglutarate-dependent enzymes in a yeast model of paraganglioma. Hum Mol Genet.

[39] Dite TA (2018). AMP-activated protein kinase selectively inhibited by the type II inhibitor SBI-0206965. J Biol Chem.

[40] TeSlaa T (2016). alpha-Ketoglutarate Accelerates the Initial Differentiation of Primed Human Pluripotent Stem Cells. Cell Metab.

[41] Chen L, Cui H (2015). Targeting Glutamine Induces Apoptosis: A Cancer Therapy Approach. Int J Mol Sci.

[42] Altman BJ, Stine ZE, Dang CV (2016). From Krebs to clinic: glutamine metabolism to cancer therapy. Nat Rev Cancer.

[43] Jin L, Alesi GN, Kang S (2016). Glutaminolysis as a target for cancer therapy. Oncogene.

[44] Seltzer MJ (2010). Inhibition of glutaminase preferentially slows growth of glioma cells with mutant IDH1. Cancer Res.

[45] Janzer A (2014). Metformin and phenformin deplete tricarboxylic acid cycle and glycolytic intermediates during cell transformation and NTPs in cancer stem cells. Proc Natl Acad Sci USA.

[46] Liu X, Romero IL, Litchfield LM, Lengyel E, Locasale JW (2016). Metformin Targets Central Carbon Metabolism and Reveals Mitochondrial Requirements in Human Cancers. Cell Metab.

[47] Alfaras I (2017). Health benefits of late-onset metformin treatment every other week in mice. NPJ Aging Mech Dis.

[48] Xiao M (2012). Inhibition of alpha-KG-dependent histone and DNA demethylases by fumarate and succinate that are accumulated in mutations of FH and SDH tumor suppressors. Genes Dev.

[49] Wu D (2018). Glucose-regulated phosphorylation of TET2 by AMPK reveals a pathway linking diabetes to cancer. Nature.

[50] Madiraju AK (2014). Metformin suppresses gluconeogenesis by inhibiting mitochondrial glycerophosphate dehydrogenase. Nature.

[51] Luengo A, Sullivan LB, Heiden MG (2014). Understanding the complex-I-ty of metformin action: limiting mitochondrial respiration to improve cancer therapy. BMC Biol.

[52] MacKenzie ED (2007). Cell-permeating alpha-ketoglutarate derivatives alleviate pseudohypoxia in succinate dehydrogenase-deficient cells. Mol Cell Biol.

[53] Murphy MP, O’Neill LAJ (2018). Krebs Cycle Reimagined: The Emerging Roles of Succinate and Itaconate as Signal Transducers. Cell.

[54] Mills EL (2016). Succinate Dehydrogenase Supports Metabolic Repurposing of Mitochondria to Drive Inflammatory Macrophages. Cell.

[55] Mills Evanna L., Pierce Kerry A., Jedrychowski Mark P., Garrity Ryan, Winther Sally, Vidoni Sara, Yoneshiro Takeshi, Spinelli Jessica B., Lu Gina Z., Kazak Lawrence, Banks Alexander S., Haigis Marcia C., Kajimura Shingo, Murphy Michael P., Gygi Steven P., Clish Clary B., Chouchani Edward T. (2018). Accumulation of succinate controls activation of adipose tissue thermogenesis. Nature.

[56] Ryan DG (2019). Coupling Krebs cycle metabolites to signalling in immunity and cancer. Nat Metab.

[57] Cuyàs Elisabet, Verdura Sara, Llorach-Pares Laura, Fernández-Arroyo Salvador, Luciano-Mateo Fedra, Cabré Noemí, Stursa Jan, Werner Lukas, Martin-Castillo Begoña, Viollet Benoit, Neuzil Jiri, Joven Jorge, Nonell-Canals Alfons, Sanchez-Martinez Melchor, Menendez Javier A. (2018). Metformin directly targets the H3K27me3 demethylase KDM6A/UTX. Aging Cell.

[58] Nelson JD, Denisenko O, Bomsztyk K (2006). Protocol for the fast chromatin immunoprecipitation (ChIP) method. Nat Protoc.

[59] Kanda Y (2013). Investigation of the freely available easy-to-use software ‘EZR’ for medical statistics. Bone Marrow Transplant.

