# Supplementary material for: Metformin activates KDM2A to reduce rRNA transcription and cell proliferation by dual regulation of AMPK activity and intracellular succinate level
Source: Sci Rep. 2019 Dec 10;9:18694. doi: 10.1038/s41598-019-55075-0 (PMC6904457; doi:10.1038/s41598-019-55075-0)
Supplement: Supplementary file 1 — Supplementary information [file 41598_2019_55075_MOESM1_ESM.pdf]

Supplementary information

Title: Metformin activates KDM2A to reduce rRNA transcription and cell proliferation by dual regulation of AMPK activity and intracellular succinate level

Authors: Yuji Tanaka[1], Akimitsu Konishi[2], Hideru Obinata[3], and Makoto Tsuneoka[1*].
Institutions: 1, Laboratory of Molecular and Cellular Biology, Faculty of Pharmacy, Takasaki University of Health and Welfare, Takasaki, Japan. 2: Department of Biochemistry, Gunma University Graduate School of Medicine, Maebashi, Japan. 3: Gunma University Initiative for Advanced Research (GIAR), Maebashi, Japan
Corresponding Author:
Correspondence to Makoto Tsuneoka, E-mail address: tsuneoka@takasaki-u.ac.jp

Materials and methods for supplementary figures
Supplementary figure S1-11
Supplementary table 1-2
Reference for Supplementary figures

Materials and methods for supplementary figures

Antibodies

Anti-phosphorylated AMPKα antibody (Thr-172), anti-AMPKα antibody, anti-phosphorylated acetyl-CoA carboxylase (ACC) antibody (Ser-79) and anti-ACC antibody were purchased (AMPK and ACC Antibody Sampler Kit, Cell Signaling; #9957). Antibody for β-actin was also purchased (Sigma, AC-15; #A5441). The anti-KDM2A antibody used in this study was described previously[1].

Primers

The primers used for the detection of the transcribed region (rDNA from +12885 to +12975 from the transcription start site; 5'-ACCTGGCGCTAAACCATTCGT-3' and 5'-GGACAAACCCTTGTGTCGAGG-3') in the ChIP assay were described previously [1]. The primers used for the detection of the AMPKα mRNA (PRKAA1) were purchased (Takara Bio; #HA165824).

Agents

Dimethyl 2-oxoglutarate (DMαKG) (TCI; #K0013) and SBI-0206965 (Sigma; #SML1540) were purchased.

Other materials and methods for experiments in the supplementary figures were same as described in the main text.

rDNA transcribed region

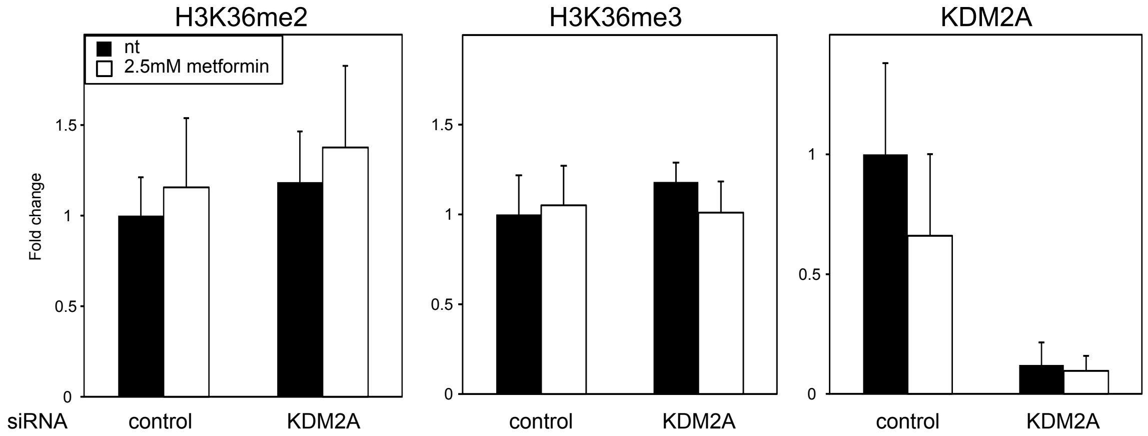

Figure S1 Metformin treatment does not reduce the levels of H3K36me2 and H3K36me3 marks in the rDNA transcribed region.
MCF-7 cells transfected with control siRNA or KDM2A siRNA were cultured with or without 2.5 mM metformin for 4 h. ChIP analyses were performed to detect H3K36me2, H3K36me3, and KDM2A in the transcribed region of rDNA. The results are expressed as fold changes of the values in various conditions to those in the cells treated with control siRNA in the absence of metformin. The experiments were performed three times (n=3), and the mean values with standard deviations are indicated.

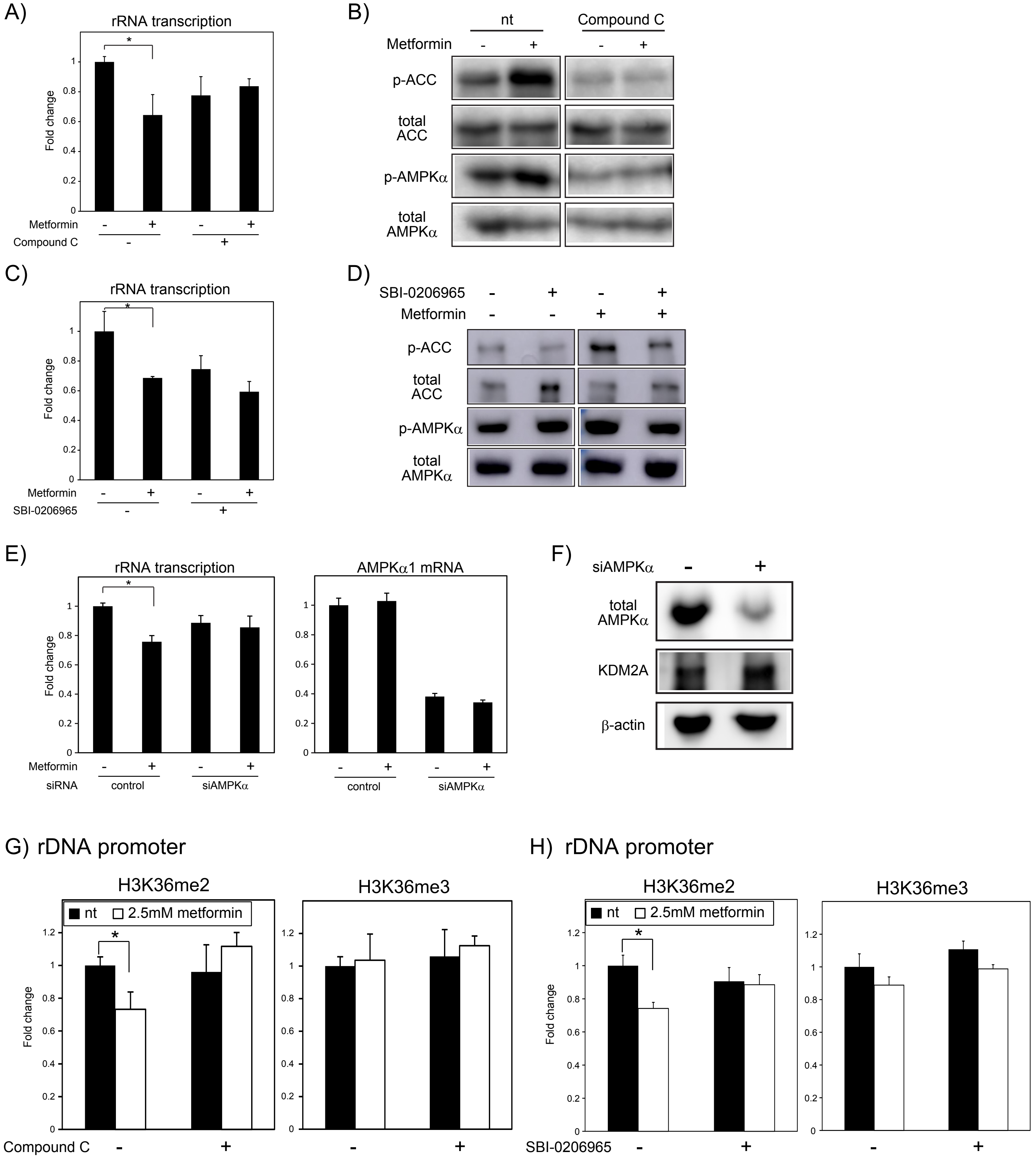
A)
rRNA transcription
Fold change
Metformin - + - +
Compound C - +
B)
nt
Compound C
Metformin - + - +
p-ACC
total ACC
p-AMPKα
total AMPKα
C)
rRNA transcription
Fold change
Metformin - + - +
SBI-0206965 - +
D)
SBI-0206965 - + - +
Metformin - - + +
p-ACC
total ACC
p-AMPKα
total AMPKα
E)
rRNA transcription
AMPKα1 mRNA
Fold change
Metformin - + - +
siRNA control siAMPKα
F)
siAMPKα - +
total AMPKα
KDM2A
β-actin
G) rDNA promoter
H3K36me2
H3K36me3
nt 2.5mM metformin
Fold change
Compound C - +
H) rDNA promoter
H3K36me2
H3K36me3
nt 2.5mM metformin
Fold change
SBI-0206965 - +

Figure S2

Figure S2 Reduction of rRNA transcription by metformin requires AMPK. (A) An inhibitor of AMPK, compound C, inhibits the reduction of rRNA transcription induced by metformin. MCF-7 cells were pre-treated with 10 μM compound C for 1 h. Then cells were cultured with or without 2.5 mM metformin for 4 h. Total RNAs were isolated and analyzed by qRT-PCR to detect rRNA transcription (pre-rRNA) and β-actin mRNA. The levels of rRNA transcription were normalized by the level of β-actin mRNA. The ratios of the values in various conditions to those without metformin and compound C are shown. The experiments were performed three times (n=3), and mean values with standard deviations are indicated. *; $P<0.05$. **(B) Compound C inhibits AMPK activity induced by metformin.** MCF-7 cells were pre-treated with 10 μM compound C for 1 h. Then cells were cultured with or without 2.5 mM metformin for 4 h. For evaluation for AMPK activity, cell lysates were collected and the levels of phosphorylated-acetyl-CoA carboxylase (ACC, Ser79) (p-ACC), known as a downstream target of AMPK, total-ACC, phosphorylated AMPKα (Thr172) (p-AMPKα) and total AMPKα were detected by immunoblotting. **(C) SBI-0206965, an inhibitor of AMPK, also inhibits the reduction of rRNA transcription induced by metformin.** MCF-7 cells were treated with or without 5 μM SBI-0206965 in presence or absence 2.5 mM metformin for 4 h. Total RNAs were isolated and analyzed by qRT-PCR to detect rRNA transcription and β-actin mRNA. The values were normalized by β-actin levels. The ratios of the values in various conditions to those without metformin and SBI-0206965 are shown. The experiments were performed three times (n=3), and mean values with standard deviations are indicated. *; $P<0.05$. **(D) SBI-0206965 inhibits AMPK activity induced by metformin.** MCF-7 cells were treated with or without 5 μM SBI-0206965 in presence or absence 2.5mM metformin for 4 h. For evaluation for AMPK activity, cell lysates were collected and the levels of p-ACC, total-ACC, p-AMPKα and total AMPKα were detected by immunoblotting. **(E) AMPK knockdown suppresses the reduction of rRNA transcription induced by metformin.** MCF-7 cells transfected with control siRNA or siRNA for AMPKα were treated with or without 2.5 mM metformin for 4 h. Total RNAs were

isolated and analyzed by qRT-PCR to detect rRNA transcription, AMPKα1 mRNA, and β-actin mRNA. The values were normalized by β-actin levels. The fold changes of the values in the various conditions to those with control siRNA without metformin are shown. The experiments were performed three times (n=3), and the mean values with standard deviations are indicated. *; $P<0.05$. **(F)** The protein levels of total AMPKα, KDM2A, and β-actin in cells transfected with siRNA for AMPKα or control siRNA were detected by immunoblotting. **(G) The treatment of compound C suppresses the reduction of H3K36me2 marks in the rDNA promoter induced by metformin.** MCF-7 cells were pre-treated with 10 μM compound C for 1 h. Then cells were cultured with or without 2.5 mM metformin for 4 h. The levels of H3K36me2 and H3K36me3 in the rDNA promoter were analyzed by ChIP assays. The results are expressed as fold changes of the values with various conditions compared to those in cells without compound C and metformin treatment. *; $P<0.05$. **(H) The treatment of SBI-0206965 suppresses the reduction of H3K36me2 marks in the rDNA promoter induced by metformin.** MCF-7 cells were treated with or without 5 μM SBI-0206965 in presence or absence 2.5 mM metformin for 4 h. The levels of H3K36me2 and H3K36me3 in the rDNA promoter were analyzed by ChIP assays. The results are expressed as fold changes of the values with various conditions to those without SBI-0206965 and metformin. *; $P<0.05$. Uncropped immunoblotting images in this figure are shown in Figure S10.

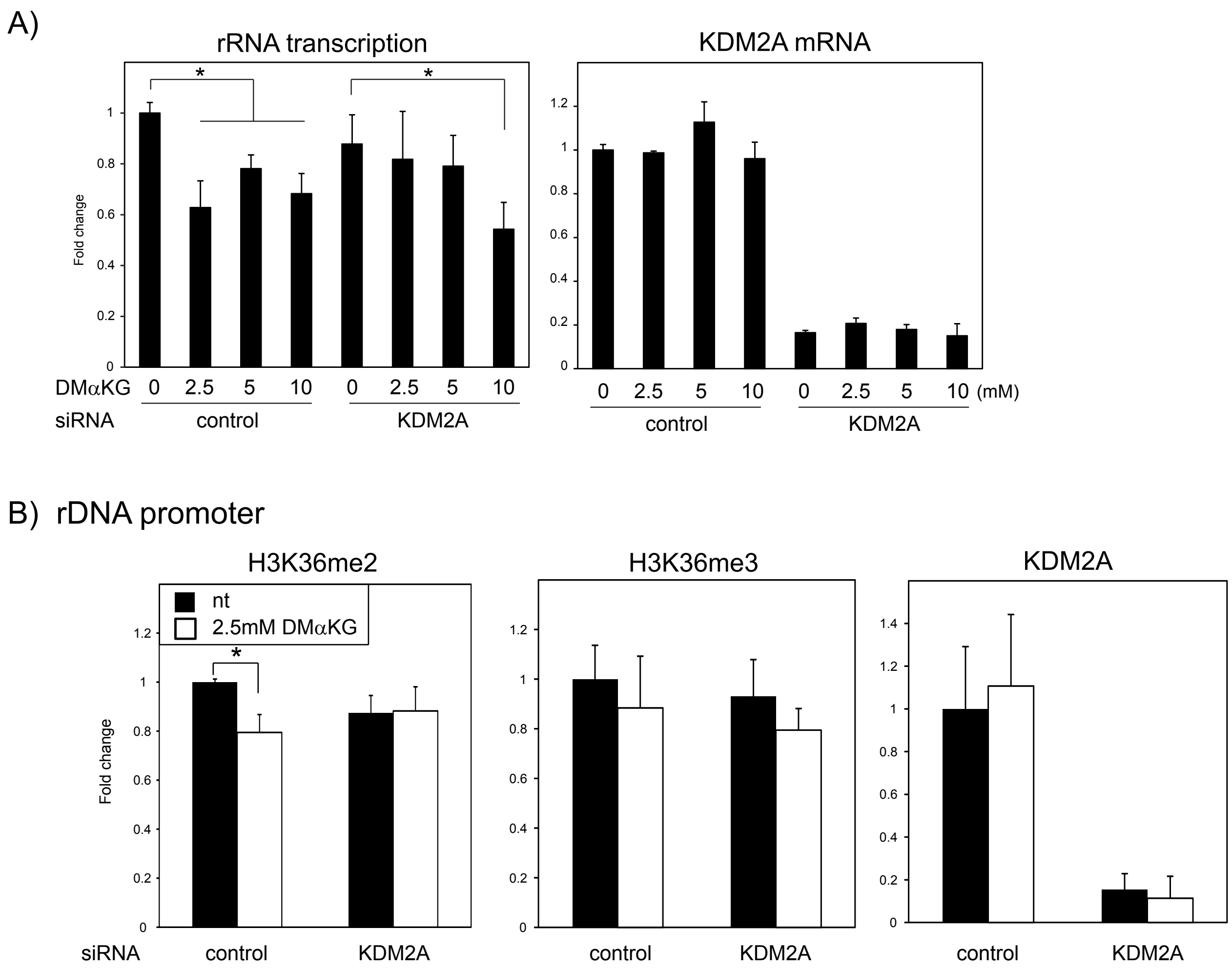

Figure S3 Cell-permeable α-ketoglutarate (DMαKG) induces KDM2A-dependent reductions of rRNA transcription and H3K36me2 marks in the rDNA promoter.
(A) KDM2A-dependent reduction of the rRNA transcription induced by DMαKG treatment. MCF-7 cells transfected with control siRNA or KDM2A siRNA were treated with a cell-permeable α-KG, dimethyl α-ketoglutarate (DMαKG), at indicated concentrations for 4 h. Total RNAs were isolated and analyzed by qRT-PCR to detect pre-rRNA (left panel), KDM2A mRNA (right panel) and β-actin mRNA. The results were normalized by the levels of β-actin mRNA. The fold changes of the values with various concentrations of DMαKG to those with control siRNA and without DMαKG are shown. **(B) DMαKG treatment induces KDM2A-dependent reduction of H3K36me2 mark in the rDNA promoter.** MCF-7 cells transfected with control siRNA or KDM2A siRNA were treated with or without 2.5 mM DMαKG for 4 h. ChIP analyses were performed to detect H3K36me2, H3K36me3, and KDM2A in the rDNA promoter. The results are expressed as fold changes of the values in various conditions compared to those in cells treated with control siRNA in the absence of DMαKG. All experiments were performed three times (n=3), and mean values with standard deviations are indicated. *P<0.05.

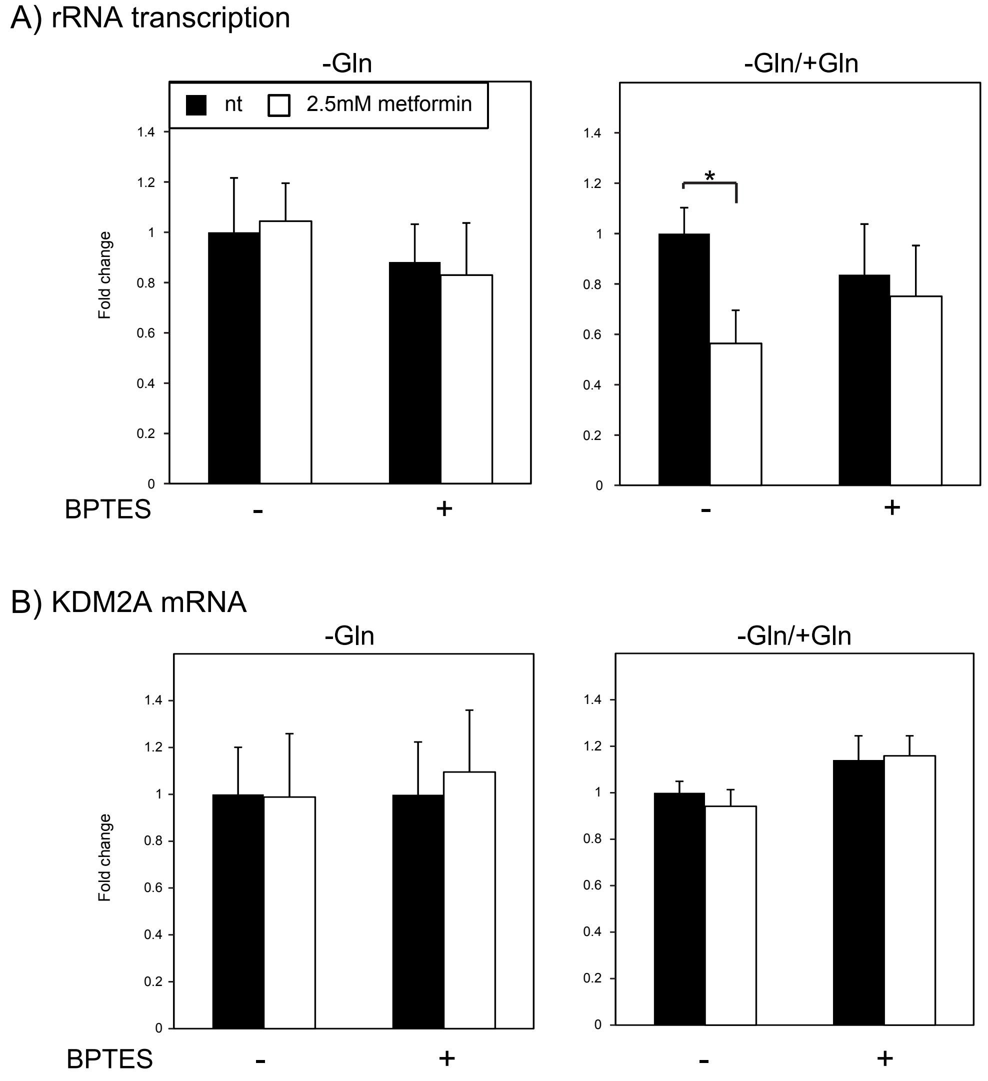

Figure S4 A glutaminase inhibitor, BPTES, suppresses the reduction of rRNA transcription induced by metformin in cells cultured in glutamine-containing medium but not in cells cultured in glutamine-free medium.

(A) Glutaminase is involved in glutaminolysis to generate α-KG from glutamine. Bis-2-(5-phenylacetamido-1,3,4-thiadiazol-2-yl) ethyl sulfide (BPTES) is an inhibitor of glutaminase. As shown in Fig. 2D, pre-treatment with BPTES abolished the reduction of rRNA transcription by metformin. To test the glutamine-dependency of the abolishment, MCF-7 cells were pre-cultured in the presence or absence of 2 μM BPTES for 16 h, and then treated with 2.5 mM metformin for 4 h in glutamine-free medium (-Gln) or glutamine-free medium with added glutamine (-Gln/+Gln) medium. The total RNAs were isolated from cells, and the levels of pre-rRNA (rRNA transcription) and β-actin mRNA were measured by qRT-PCR. The results are shown as fold changes of the values in cells in various conditions compared to those in cells treated without metformin and BPTES. (B) The expressions of KDM2A and β-actin mRNAs in (A) were detected. The results are shown as fold changes of the values of cells cultured in various conditions compared to those of cells cultured in glutamine-free medium (-Gln) without metformin. The experiments were performed three times, and the mean values with standard deviations are indicated. *; $P<0.05$.

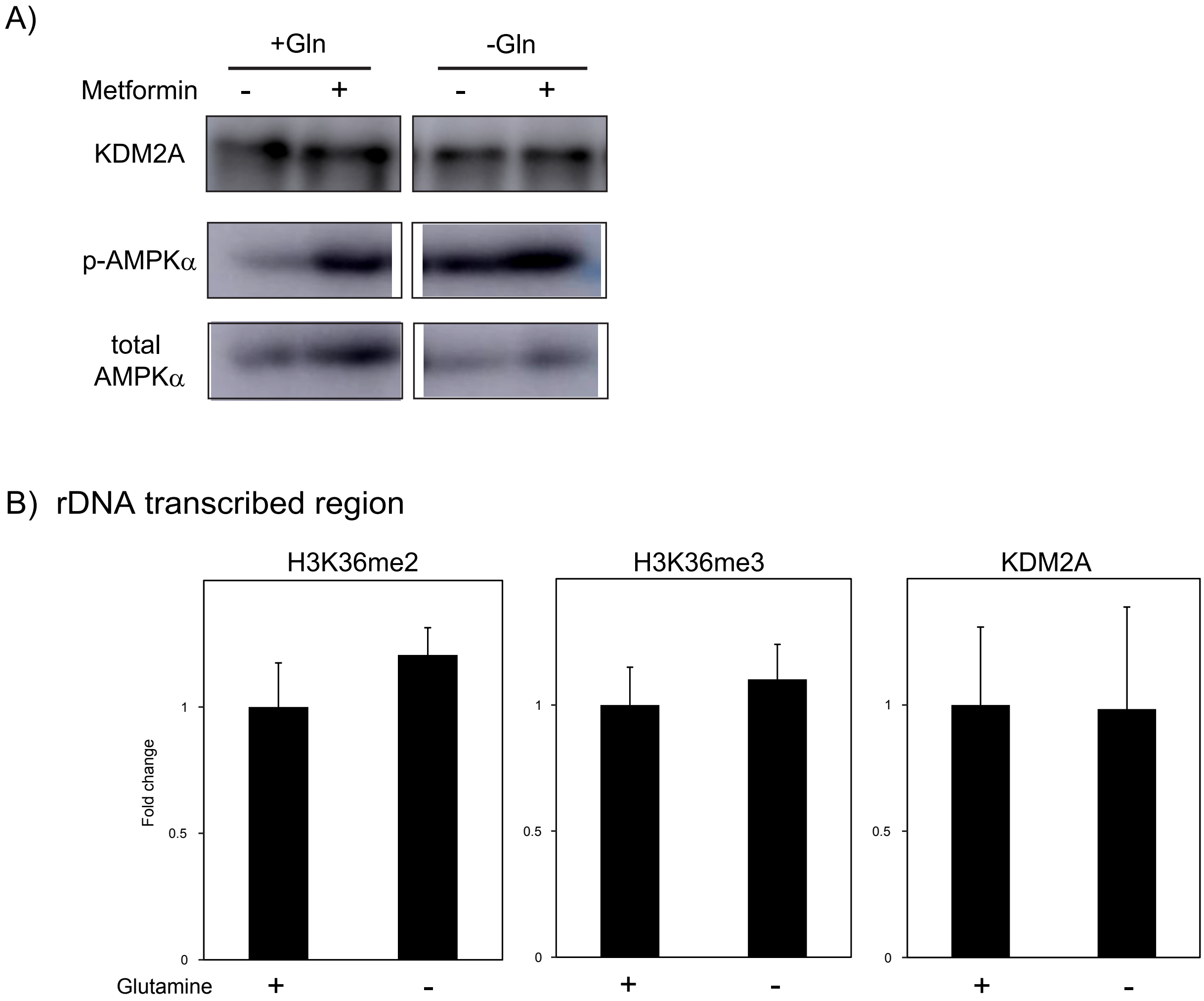

Figure S5 Glutamine-depletion alone activates AMPK but does not reduce H3K36me2 marks in the rDNA transcribed region.
(A) Activation of AMPK in cells cultured in glutamine-free medium. Cells were cultured with or without 2.5 mM metformin in glutamine-free medium or glutamine-containing medium for 4 h, and cell lysates were immunoblotted to detect KDM2A, the phosphorylated-AMPKα (Thr172), and total AMPKα in cells. The uncropped images were shown in Supplementary Figure S11. **(B) Glutamine-depletion alone did not reduce H3K36me2 marks in the rDNA promoter.** MCF-7 cells cultured in glutamine-containing medium (+Gln) or glutamine-free medium (–Gln) for 4 h. The levels of H3K36me2, H3K36me3, and KDM2A in the rDNA promoter were analyzed by ChIP assays. The results are expressed as fold changes of the values in various conditions relative to those of the cells cultured in glutamine-containing medium (+Gln). The experiments were performed three times (n=3), and the mean values with standard deviations are indicated.

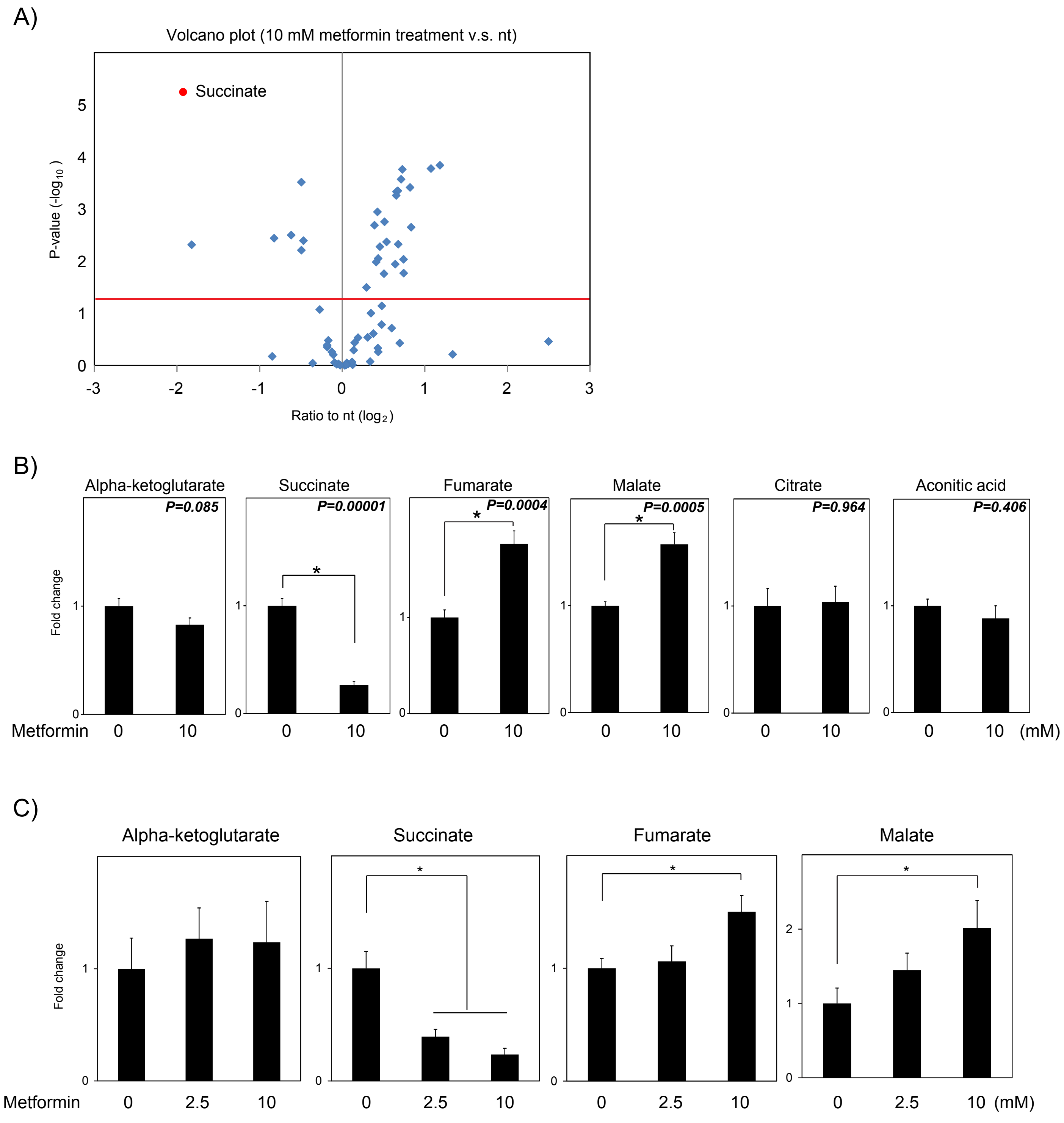

Figure S6 Metformin treatment with 10 mM for 4 h decreases the succinate level in cells. (A) Volcano plot of metabolites in cells treated with 10 mM metformin. MCF-7 cells were treated with or without 10 mM metformin for 4 h. The metabolites of cells were analyzed by LC-MS/MS. The results are shown as a volcano plot expressed with the ratios and p-values calculated from comparison of the results in cells treated with 10 mM metformin and without metformin. The values for succinate are shown as a red circle. The red line indicates where $p = 0.05$ with points above the line having $p < 0.05$. The details of these data are shown in Supplementary Table 1. **(B) Levels of TCA cycle intermediates in cells treated with 10 mM metformin.** The levels of α-KG, succinate, fumarate, malate, citrate, and aconitic acid in cells treated with 10 mM metformin are shown as the fold changes of those without metformin. The experiments were performed three times (n=3), and the mean values with standard deviations and p-values are indicated. *; $P<0.05$. **(C) Levels of TCA cycle intermediates after metformin treatment were confirmed by LC-MS/MS with a different LC method.** The experiments were performed by methods basically the same as those described in Figs. 4 and S6A, except that a MastroTM SP column was used to separate metabolites. The ratios of the levels of α-KG, succinate, fumarate, malate, citrate, and aconitic acid in cells treated with 2.5 and 10 mM metformin to those in untreated cells are shown. The experiments were performed three times (n=3), the mean values were calculated, and the fold changes are shown with standard deviations. *; $P<0.05$.

rDNA transcribed region

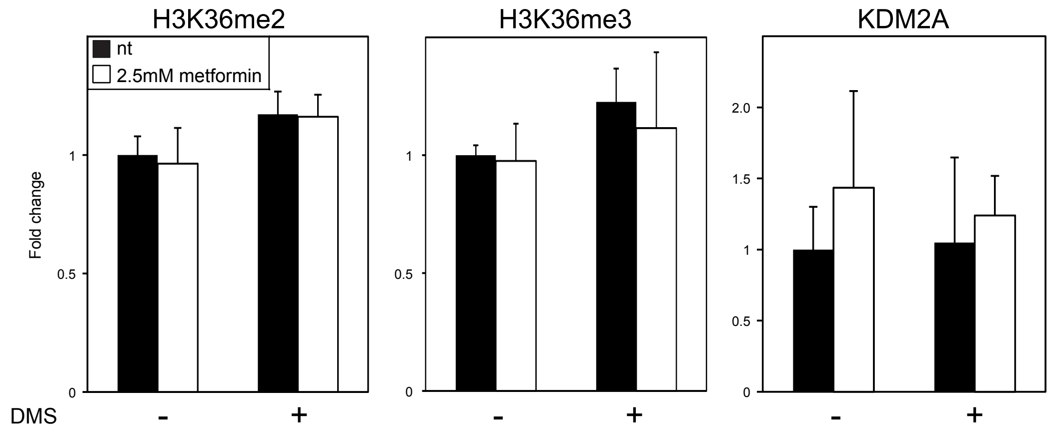

Figure S7 Treatments with metformin and DMS did not affect the levels of H3K36me2 and H3K36me3 in the rDNA transcribed region. MCF-7 cells were treated with or without 2.5 mM metformin in the presence or absence of 5 mM dimethyl succinate (DMS) for 4 h. The levels of KDM2A, H3K36me2, and H3K36me3 in the rDNA transcribed region were analyzed by ChIP assays. The fold changes of the values with various conditions to those without DMS and metformin are calculated. The experiments were performed three times (n=3), and the mean values with standard deviations are shown.

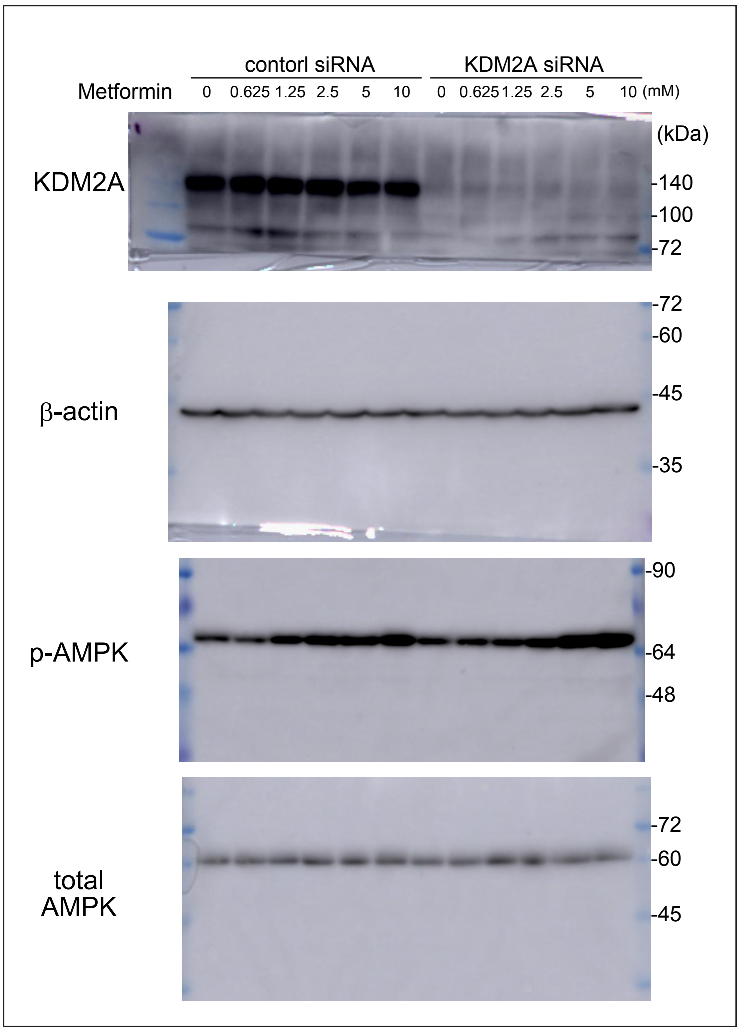

Figure S8 Uncropped immunoblot images for Figure 1B.

For figure 4B

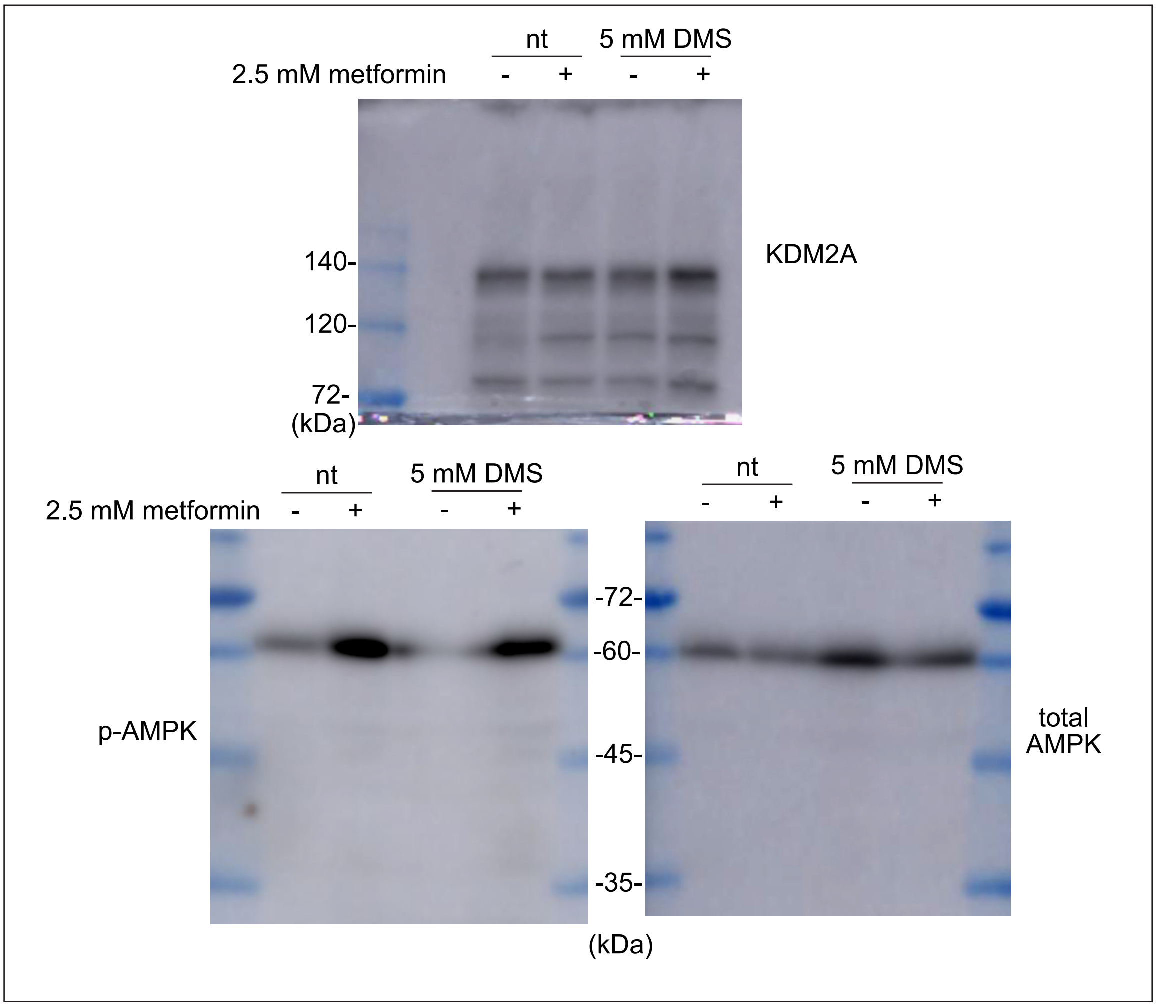

Figure S9 Uncropped immunoblot images for Figure 4B

For figure S2B

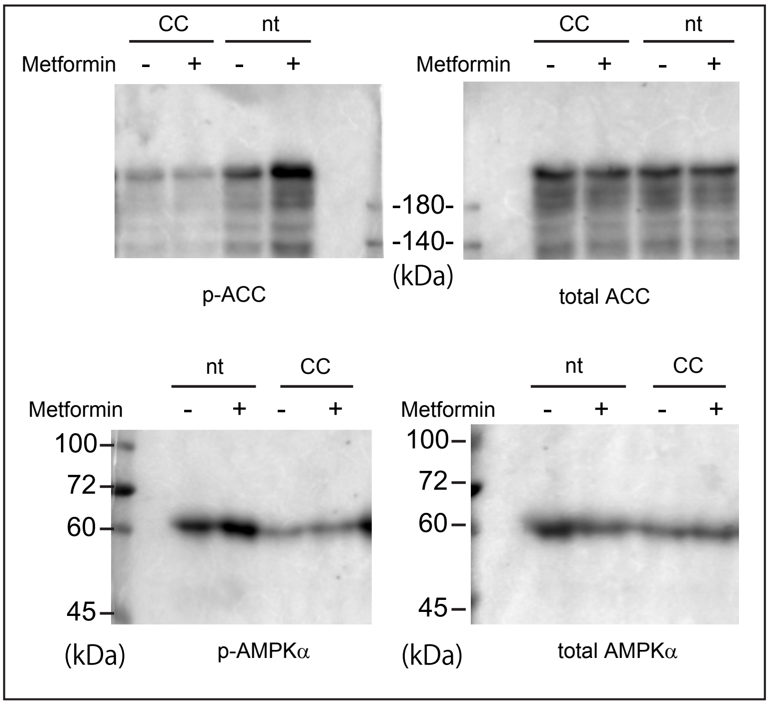

For figure S2F

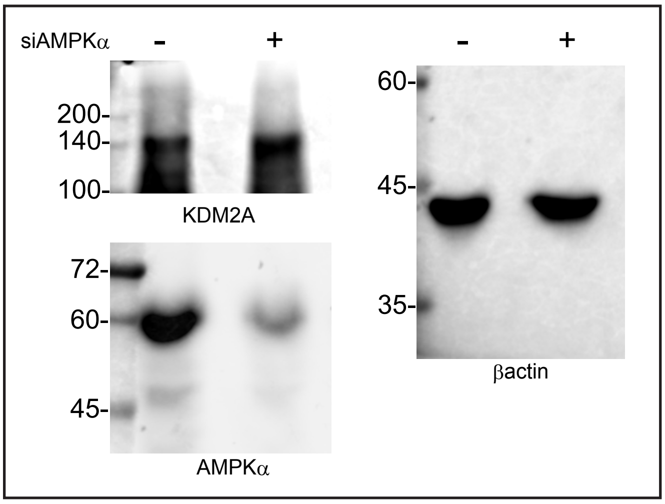

For Fig.S2D

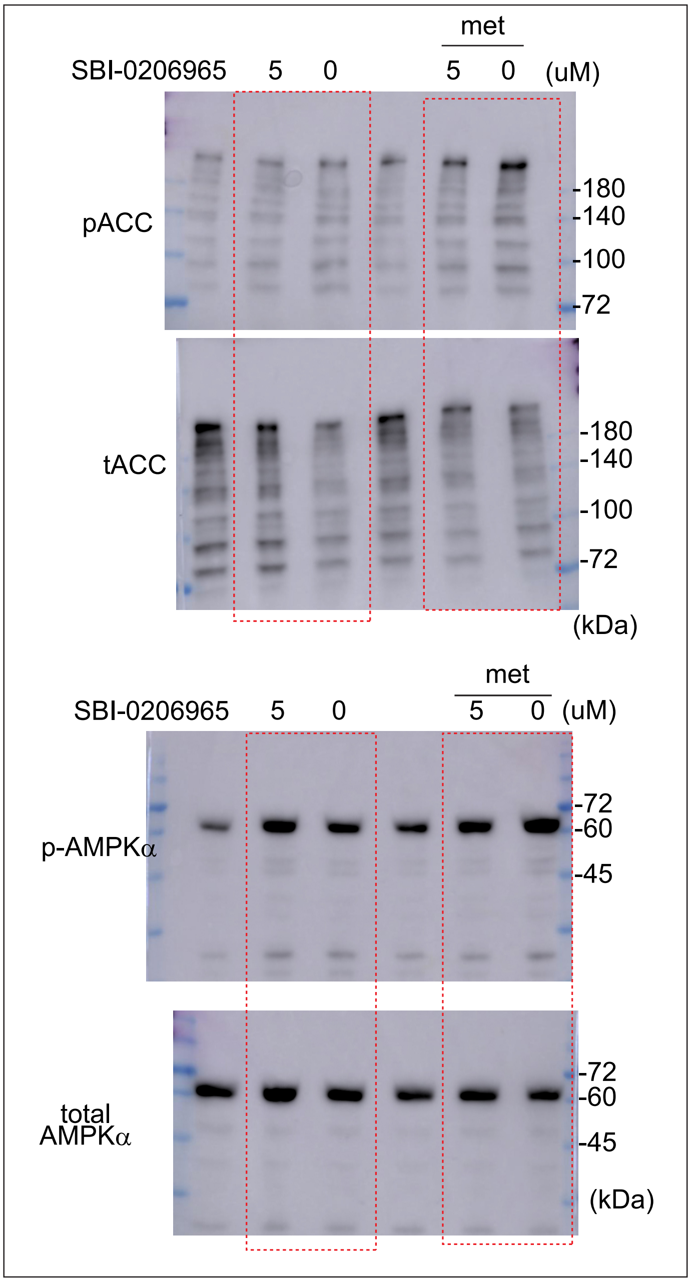

Figure S10 Uncropped immunoblot images for Supplementary Figure S2.

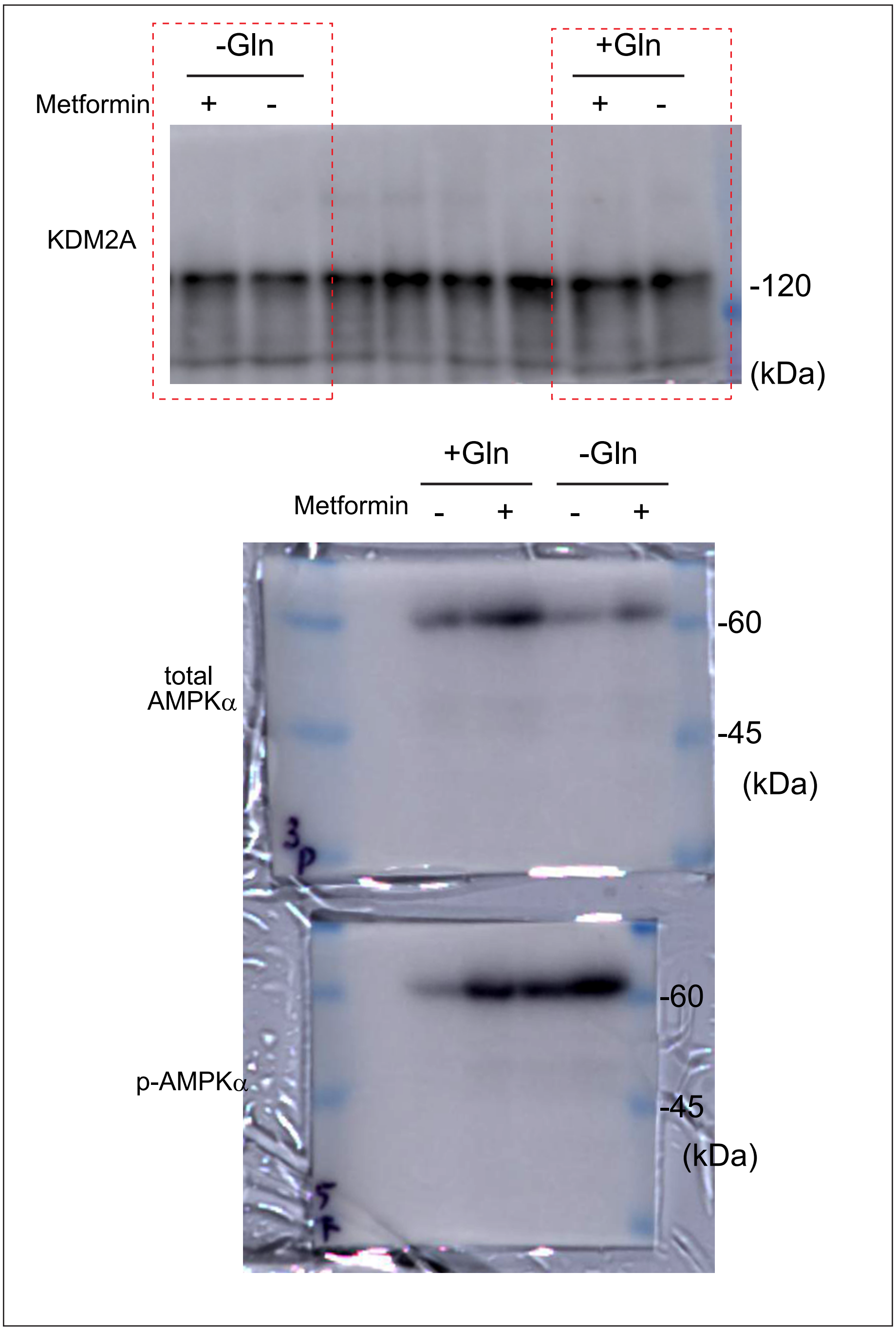

Figure. S11
Uncropped immunoblot images for Supplementary Figure S5A.

		ratio to nt		P-value(vs nt)	
Mebalolite name	KEGG number	Met 2.5 mM	Met 10 mM	Met 2.5 mM	Met 10 mM
NAD	C00003	1.04	1.10	0.89426	0.50818
FAD	C00016	0.95	0.96	0.93097	0.94635
Adenosine monophosphate	C00020	1.12	1.68	0.76294	0.01695
S-Adenosylhomocysteine	C00021	1.69	0.78	0.41414	0.90363
Pyruvic acid	C00022	1.08	1.40	0.85289	0.07207
Glutamic acid	C00025	0.83	0.71	0.00499	0.00030
2-Ketoglutaric acid	C00026	0.98	0.83	0.96478	0.08479
Glycine	C00037	0.92	1.04	0.66261	0.89097
Alanine	C00041	0.96	1.23	0.81300	0.03183
Succinic acid	C00042	0.49	0.26	0.00005	0.00001
Aspartic acid	C00049	0.61	0.57	0.00633	0.00363
Cytidine monophosphate	C00055	0.93	1.09	0.98180	0.96993
Arginine	C00062	1.11	1.45	0.46020	0.00428
Glutamine	C00064	1.06	1.35	0.52449	0.00114
Serine	C00065	1.07	1.39	0.93305	0.16449
Methionine	C00073	1.13	1.60	0.24004	0.00045
Ornitine	C00077	1.17	1.64	0.12440	0.00027
Tryptophan	C00078	1.99	2.53	0.80562	0.61349
Phenylalanine	C00079	1.17	1.66	0.10892	0.00017
Tyrosine	C00082	1.24	1.62	0.84601	0.37270
Cysteine	C00097	1.12	1.26	0.96576	0.83926
Choline	C00114	1.34	1.67	0.13023	0.00923
Fumaric acid	C00122	1.26	1.77	0.06449	0.00039
Leucine	C00123	1.27	1.78	0.17665	0.00224
Oxidized glutathione	C00127	0.88	0.98	0.55609	0.98716
Histidine	C00135	1.04	1.37	0.85547	0.00526
Guanosine monophosphate	C00144	n.d	0.91	n.d	0.69072
Adenine	C00147	1.19	1.52	0.76198	0.19236
Proline	C00148	0.98	1.09	0.99398	0.85493
Malic acid	C00149	1.15	1.57	0.18748	0.00055
Asparagine	C00152	0.87	1.05	0.67901	0.93982
Niacinamide	C00153	0.96	0.93	0.85288	0.62366
Citric acid	C00158	1.12	1.04	0.65967	0.96362
Valine	C00183	1.11	1.58	0.33846	0.00046
Lactic acid	C00186	1.40	2.11	0.02770	0.00017
Threonine	C00188	1.04	1.31	0.74898	0.00205
Adenosine	C00212	1.00	0.56	0.99999	0.66892
Nicotinic acid	C00253	0.95	0.97	0.81852	0.93353
Creatine	C00300	1.09	1.35	0.50819	0.00889
Citicoline	C00307	0.97	1.24	0.97902	0.28985
Citrulline	C00327	0.97	1.56	0.96724	0.01143
Kynurenine	C00328	1.10	1.43	0.37289	0.00176
4-Aminobutyric acid	C00334	0.73	0.71	0.00824	0.00617
Uric acid	C00366	1.00	1.02	0.99959	0.98977
Xanthine	C00385	0.86	0.92	0.21773	0.54928
Guanosine	C00387	0.95	0.88	0.83560	0.44352
Isoleucine	C00407	1.18	1.60	0.34512	0.00472
Aconitic acid	C00417	0.98	0.88	0.95588	0.40648
Cytidine	C00475	2.18	5.66	0.92261	0.34578
Carnitine	C00487	1.15	1.42	0.37678	0.01741
cAMP	C00575	0.90	0.94	0.73735	0.88179
5-Glutamylcysteine	C00669	0.85	0.65	0.11226	0.00316
Creatinine	C00791	1.04	1.14	0.90887	0.29238
Pantothenic acid	C00864	0.96	1.02	0.96540	0.98751
cCMP	C00941	1.07	1.33	0.60474	0.01037
4-Hydroxyproline	C01157	0.92	1.11	0.55044	0.36570
Cysteamine	C01678	1.04	1.04	0.90399	0.92541
Cystathionine	C02291	0.83	0.89	0.10782	0.33045
2-Aminobutyric acid	C02356	1.03	1.27	0.96033	0.09927
Acetylcarnitine	C02571	1.05	1.35	0.98299	0.46584
Methionine sulfoxide	C02989	1.03	1.30	0.98583	0.24501
Argininosuccinic acid	C03406	1.41	2.27	0.04154	0.00015
Asymmetric dimethylarginine	C03626	0.64	0.28	0.08529	0.00485
Symmetric dimethylarginine	C03626	0.94	1.35	0.97837	0.54883
Ophthalmic acid	C21016	0.91	0.72	0.25013	0.00405

Supplementary table 1

Supplementary Table 1 Metabolites levels at four hours after metformin treatment.

After cells were treated with 0, 2.5, or 10 mM metformin for 4 h, the levels of 65 metabolites were quantitated by LC-MS/MS. The experiments were performed three times (n=3), and the mean values and *p*-values (from Tukey-test) were calculated. The ratios of all metabolite (65 metabolites) in cells with 4-hour metformin treatment against those in cells without metformin are shown with *p*-values. The orange rows show the metabolites that were significantly increased by metformin treatment (P<0.05). The blue rows show the metabolites significantly reduced by metformin (P<0.05).

Decreased by 2.5 mM met in 4h (P<0.05)

Metabolite	KEGG no.	Fold change
**Succinic acid**	**C00042**	**0.49**
Aspartic acid	C00049	0.61
4-Aminobutyric acid	C00334	0.73
Glutamic acid	C00025	0.83

Increased by 2.5 mM met in 4h (P<0.05)

Lactic acid	C00186	1.40
Argininosuccinic acid	C03406	1.41

Decreased by 10 mM met in 4h (P<0.05)

**Succinic acid**	**C00042**	**0.26**
Asymmetric dimethylarginine	C03626	0.28
Aspartic acid	C00049	0.57
5-Glutamylcysteine	C00669	0.65
4-Aminobutyric acid	C00334	0.71
Glutamic acid	C00025	0.71
Ophthalmic acid	C21016	0.72

Increased by 10 mM met in 4h (P<0.05)

Alanine	C00041	1.23
Threonine	C00188	1.31
cCMP	C00941	1.33
Glutamine	C00064	1.35
Creatine	C00300	1.35
Histidine	C00135	1.37
Carnitine	C00487	1.42
Kynurenine	C00328	1.43
Arginine	C00062	1.45
Citrulline	C00327	1.56
Malic acid	C00149	1.57
Valine	C00183	1.58
Methionine	C00073	1.60
Isoleucine	C00407	1.60
Ornitine	C00077	1.64
Phenylalanine	C00079	1.66
Choline	C00114	1.67
Adenosine monophosphate	C00020	1.68
Fumaric acid	C00122	1.77
Leucine	C00123	1.78
Lactic acid	C00186	2.11
Argininosuccinic acid	C03406	2.27

Supplementary table 2

Supplementary Table 2 Metabolites significantly changed by 4-hour metformin treatment. The metabolites significantly changed by the 4-hour metformin treatments were extracted from Supplementary Table 1. Four metabolites were decreased and Two metabolites were increased by 2.5 mM metformin treatment for 4 h with statistical significance (p<0.05). Seven metabolites were decreased and 22 metabolites were increased by 10 mM metformin treatment for 4 h with statistically significant (p<0.05).

Reference for Supplementary figures

1. Tanaka, Y. *et al.* JmjC enzyme KDM2A is a regulator of rRNA transcription in response to starvation. *EMBO J* **29**, 1510-1522 (2010).
